# Predictive value of HDL function in patients with coronary artery disease: relationship with coronary plaque characteristics and clinical events

**DOI:** 10.1080/07853890.2022.2063374

**Published:** 2022-04-19

**Authors:** Marco Magnoni, Daniele Andreini, Angela Pirillo, Patrizia Uboldi, Roberto Latini, Alberico L. Catapano, Aldo P. Maggioni, Giuseppe D. Norata, on behalf of the CAPIRE Study Group

**Affiliations:** a IRCCS Ospedale San Raffaele, Milan, Italy; b IRCCS, Centro Cardiologico Monzino, Milan, Italy; c Department of Biomedical and Clinical Sciences “Luigi Sacco”, University of Milan, Milan, Italy; d Centro SISA per lo Studio dell’Aterosclerosi, Ospedale Bassini, Balsamo, Italy; e IRCSS Multimedica, Milan, Italy; f Department of Excellence of Pharmacological and Biomolecular Sciences, Università Degli Studi di Milano, Milan, Italy; g Department of Cardiovascular Medicine, IRCCS – Istituto di Ricerche Farmacologiche “Mario Negri”, Milan, Italy; h Heart Care Foundation ANMCO Research Center, Florence, Italy

**Keywords:** Cholesterol efflux capacity, SR-BI, atherosclerotic plaque volume, coronary artery disease

## Abstract

**Background:**

HDL is endowed with several metabolic, vascular, and immunoinflammatory protective functions. Among them, a key property is to promote reverse cholesterol transport from cells back to the liver. The aim of this study was to estimate the association of scavenger receptor class B type I (SR-BI)- and ATP binding cassette transporter A1 (ABCA1)-mediated cholesterol efflux (the two major routes for cholesterol efflux to HDL) with the presence, extent, and severity of coronary artery disease (CAD), vascular wall remodelling processes, coronary plaque characteristics, and the incidence of myocardial infarction in the different subgroups of patients from the CAPIRE study.

**Methods:**

Patients (*n* = 525) from the CAPIRE study were divided into two groups: low-risk factors (RF), with 0–1 RF (*n* = 263), and multiple-RF, with ≥2 RFs; within each group, subjects were classified as no-CAD or CAD based on the segment involvement score (SIS) evaluated by coronary computed tomography angiography (SIS = 0 and SIS > 5, respectively). SR-BI- and ABCA1-mediated cholesterol efflux were measured using the plasma of all patients.

**Results:**

SR-BI-mediated cholesterol efflux was significantly reduced in patients with CAD in both the low-RF and multiple-RF groups, whereas ABCA1-mediated cholesterol efflux was similar among all groups. In CAD patients, multivariable analysis showed that SR-BI-mediated cholesterol efflux <25^th^ percentile predicted cardiovascular outcome (odds ratio 4.1; 95% CI: 1.3–13.7; *p* = .019), whereas ABCA-1-mediated cholesterol efflux and HDL-C levels significantly did not. Despite this finding, reduced SR-BI-mediated cholesterol efflux was not associated with changes in high-risk plaque features or changes in the prevalence of elevated total, non-calcified, and low-attenuation plaque volume.

**Conclusion:**

SR-BI-mediated cholesterol efflux capacity is lower in patients with diffuse coronary atherosclerosis. In addition, a lower SR-BI-mediated cholesterol efflux capacity is associated with the worst clinical outcomes in patients with CAD, independently of atherosclerotic plaque features.

**Key Messages**
- Increased cholesterol efflux capacity, an estimate of HDL function, is associated with a reduced CVD risk, regardless of HDL-C levels.
- HDL-C levels are significantly lower in patients with CAD.
- Lower SR-BI-mediated cholesterol efflux capacity is observed in patients with diffuse coronary atherosclerosis and is associated with the worst clinical outcomes in patients with CAD, independently of atherosclerotic plaque features.

## Introduction

Coronary artery disease (CAD) is a leading cause of death, despite continuous improvements in prevention and treatment [1]. The risk of coronary events is conventionally calculated by multifactorial stratification methods that integrate traditional risk factors (RFs) [2,3]. Among different RFs, epidemiological data highlighted an inverse relationship between high-density lipoprotein cholesterol (HDL-C) levels and the incidence of cardiovascular disease (CVD), at least for values up to 80–90 mg/dL [4,5], while at higher levels a U-shaped association has been demonstrated, with extremely high HDL-C levels being associated with an increased CVD risk [6]. Uncertainty on the causal role of HDL in CVD is still elevated [6–8]; a possible explanation is that HDL-C levels do not always provide information on the functionality of HDL particles in a specific setting.

Indeed, HDL is endowed with several metabolic, vascular, and immunoinflammatory protective functions [9,10]. Among them, a key property is to promote reverse cholesterol transport, i.e. cholesterol efflux from cells back to the liver [11]. This evolutionarily conserved mechanism initiates with the ATP binding cassette transporter A1 (ABCA1)-mediated unidirectional export of cholesterol and phospholipids from cells to lipid-poor apolipoprotein A-I (the main apolipoprotein of HDL), leading to the formation of nascent, discoidal pre-ß-HDL particles. Next, two transporters, namely scavenger receptor class B type I (SR-BI, which mediates a bidirectional flux of free cholesterol between cells and HDL) and ABCG1 mediate further cholesterol efflux and contribute to HDL maturation and the generation of large, spherical HDL particles. The assessment of cholesterol efflux capacity (CEC) has been adopted as an estimate of HDL function [11]; of note, HDL particles exhibit high levels of structural and compositional heterogeneity, and phospholipid content and composition are major factors determining the HDL cholesterol efflux capacity [12,13]. Several studies have investigated the association between CEC and the incidence of cardiovascular events in the general population, and many, but not all, observed that an increased CEC is associated with a reduced CVD risk, regardless of HDL-C concentration [14–17]. This association, however, is not as robust in patients with reduced kidney function [18] or end-stage renal disease [19] in whom, on the other hand, HDL-C levels predict disease progression [18,19], suggesting that HDL function can be a good predictor of CVD, at least in initially healthy individuals without clinically manifest CVD.

Early discrimination of subjects with established CVD but no clinical symptoms is an emerging area of discussion where the possibility of strengthening traditional RF-based CV risk assessment by including a direct estimation of coronary atherosclerosis is an intriguing option. Coronary computed tomography angiography (CCTA) is a comprehensive non-invasive diagnostic test that provides information about the presence, extent, and severity of CAD, vascular wall remodelling processes, and plaque characteristics. It enables the identification of subjects with normal coronary arteries (high negative predictive value) or with subclinical disease, and it better defines the global atherosclerotic process [20]. Furthermore, CCTA allows for identifying patients with unexpected diffuse CAD despite a low RF profile, as well as those who, despite the presence of multiple RFs, develop only mild or no coronary atherosclerosis [21–23]. These extreme “outlier” populations have been specifically investigated in the CAPIRE (Coronary Atherosclerosis in outlier subjects: Protective and novel Individual Risk factor Evaluation) study [24], a hypothesis-generating study aimed at exploring protective and susceptibility factors of CAD to identify high-risk subjects who may benefit from a more personalised prevention strategy. 

The aims of this study were therefore to estimate HDL CEC, discriminate between cholesterol efflux to nascent and mature HDL, and its association with coronary artery characteristics and the incidence of myocardial infarction (MI) in the population from the CAPIRE study.

## Methods

### CAPIRE study

The CAPIRE (Coronary Atherosclerosis in Outlier Subjects: Protective and Individual Risk Factor Evaluation) study (NCT02157662) is part of the GISSI Outlier Project; it is a prospective, observational, international multicenter study involving a cross-sectional comparison of several variables (clinical, imaging, and biomolecular) with a 10-year follow-up [24]; the data presented here refer to a 5-year follow-up period.

### Study population

For this study, 525 consecutive patients aged 45–75 years without acute coronary syndrome and with normal left ventricular ejection fraction were recruited. Participants underwent 64-slice (or superior) CCTA for suspected CAD in the outpatient clinics of the 11 centres involved in the study, and, based on CCTA results and risk profile, they were divided into four groups, according to pre-specified criteria:

- Low-RF/no-CAD: subjects with 0–1 RF (with the exclusion of patients with type 1 or type 2 diabetes mellitus as single RF) and no CAD;
- Low-RF/CAD: subjects with 0–1 RF (with the exclusion of patients with type 1 or type 2 diabetes mellitus as single RF) and diffuse CAD extended to >5 of the 16 segments defined by the American Heart Association (AHA) classification [25];
- Multiple-RF/no-CAD/: subjects with ≥ 2 RFs and no CAD;
- Multiple-RF/CAD: subjects with ≥2 RFs and diffuse CAD extended to >5 segments.

Patients with low CCTA quality control criteria, or reporting previous cardiovascular events (including acute MI, unstable or chronic stable angina, percutaneous or surgical coronary revascularization, and heart failure), dilated cardiomyopathy, obstructive hypertrophic cardiomyopathy, atrial fibrillation, myocarditis, inflammatory vascular disease, acute or chronic peripheral vascular disease, active inflammatory, or neoplastic disease were not enrolled in the study. The protocol was approved by the local Ethical Committee of each participant site and all patients provided informed consent. The list of participating centres is provided in the Appendix section.

Risk factors included family history of CAD (history of early manifestations of CAD in first-degree relatives, <55 years old for men and <65 years old for women), systemic hypertension (history of arterial hypertension, ongoing antihypertensive treatment, or recent observation of blood pressure values >140/90 mmHg), hypercholesterolaemia (total cholesterol >200 mg/dl or <200 mg/dl if under lipid-lowering therapy), diabetes mellitus (fasting plasma blood glucose levels >126 mg/dL, or 2-h values in the oral glucose tolerance test ≥200 mg/dL, or isolated elevation of glycated haemoglobin ≥6.5%, or current use of insulin or oral hypoglycaemic agents), and cigarette smoking (current smoker or < 1-year abstention) [26].

Physical examination, anamnestic records, and laboratory tests provided by the participants or documented before CCTA were used to define an individual’s risk factors. Lipid profile and metabolic parameters were evaluated also in a centralised core laboratory to validate the local assessment of RFs such as diabetes or hypercholesterolaemia.

### Laboratory analysis

A peripheral venous blood sample was collected from each patient at the enrolment. The samples were immediately processed to obtain separate aliquots of whole blood, plasma, and serum and stored at −70 °C in a dedicated biological bank (HCF blood bank, located at SATURNE-1, Mario Negri Institute of Pharmacological Research, Milan).

Circulating biomarkers were measured in a central laboratory, in a single batch, by personnel unaware of patients’ characteristics. High-sensitivity C-reactive protein was measured with an automatic immunoturbidimetric method (Beckman-Coulter, Galway, Ireland). Serum creatinine and lipids were measured with standard, automated laboratory methods.

### HDL-mediated cholesterol efflux evaluation

To evaluate SR-BI-mediated cholesterol efflux, Fu5AH cells were grown to subconfluence, then incubated for 24 h with DMEM containing 5% FCS, ^3^H-cholesterol (1 µCi/ml), and 2 µg/ml ACAT inhibitor Sandoz 58-035. After washing, cells were incubated overnight in fresh DMEM containing 0.2% BSA and 2 µg/ml Sandoz 58-035. For efflux, cells were incubated with 1.5% plasma diluted in a serum-free medium for 4 h. The media were collected, centrifuged, and aliquots were used for liquid scintillation counting. Cell monolayers were lysed with 0.1 N NaOH and aliquots were used for liquid scintillation counting. The efflux of ^3^H‐cholesterol was calculated as the ratio of radioactivity released into the medium to the total (medium-plus intracellular) radioactivity. To correct for inter-assay variation across plates, a pooled plasma control from two healthy volunteers was included in each plate, and values for plasma samples from patients were normalised to this pooled value in all analyses. Intra- and inter-assay coefficients of variation for SR-BI-mediated cholesterol efflux were 4.7% and 14.3%, respectively.

To investigate the role of ABCA1 in cholesterol efflux, J774 cells were labelled for 24 h with MEM containing 50 µg/ml AcLDL, ^3^H-cholesterol (1 µCi/ml), and 2 µg/ml Sandoz 58-035. After washing, cells were incubated for 18 h in MEM containing 0.2% BSA, 0.3 mM 8-Br-cAMP, and 2 µg/ml Sandoz 58-035. For efflux, cells were incubated with 1% plasma of individual patients diluted in serum-free medium for 4 h. Samples were processed as described above.

### Coronary CTA analysis

All CCTA scans were transferred to the CCTA Core Lab (Centro Cardiologico Monzino, Milano) for a central blinded analysis of coronary angiograms. Coronary plaques were defined as structures of at least 1 mm^2^ area within and/or adjacent to artery lumen, clearly distinguishable from the vessel lumen, and surrounded by pericardial tissue; tissue with signal intensity below −40HU was considered a pericardial fat and excluded from the analysis. Coronary arteries were referred to as normal in the absence of atherosclerotic plaque (including focal and eccentric calcified plaques) in each segment.

Details on the evaluation of high-risk plaque features (HPFs) have been reported previously [27]. Plaque consistency was evaluated using Hounsfield Unit (HU); low-attenuation plaque volume and non-calcified plaque volume were defined as <30 HU and <150 HU, respectively, and expressed in mm^3^ [28]. Total plaque volume was evaluated and reported in mm^3^. All plaque volumes have also been evaluated as qualitative dichotomous variables using the higher quartile as cut-off on a per-patient basis.

### Five-years follow-up findings

Clinical visits were scheduled every 12 months, with structured phone interviews planned every 6 months. All clinical events were recorded and validated centrally by an event committee blinded to the CCTA results. Hospital records and outsourced clinical documents were screened to confirm the information obtained. For this analysis, data on cardiac death, acute coronary syndrome (ACS), and non-urgent revascularization were recorded. The definition of these events has been reported in detail in a previous paper [27].

### Statistical analysis

Continuous variables were presented as mean ± SD or median with interquartile range (25^th^–75^th^) (for non-normal distribution). Student’s t-test for independent samples and the analysis of variance (ANOVA) for repeated measurements was used to compare continuous normally distributed variables; Mann-Whitney U tests for independent samples and Wilcoxon test for repeated measurements were used for non-normal distributions. The proportion of categorical variables was compared using a Chi-square analysis or Fisher’s exact test. Values of *p* < .05 were considered statistically significant. Odds ratios (ORs) with 95% confidence intervals (CIs) and estimated hazard ratios (HR) with 95% CIs were presented for each class of the variables that were significant in the univariate analysis. The event-free survival curves were analysed using the Kaplan-Meier method and compared using the log-rank test. Cox regression analysis was performed to identify the independent predictors of clinical outcomes. The multivariable model was created including all the variables with a probability value of <0.05 in the univariate analysis.

Statistical analysis was performed using SAS (version 11, SAS Institute Inc. 2013 Cary, North Carolina) and JMP software (version 11.0.0, SAS Institute Inc., Cary, North Carolina, USA). Comparisons of areas under the ROC curve were performed using MedCalc Statistical Software (version 12.3.0, MedCalc Software bvba2013, Ostend, Belgium).

## Results

**Figure 1.**
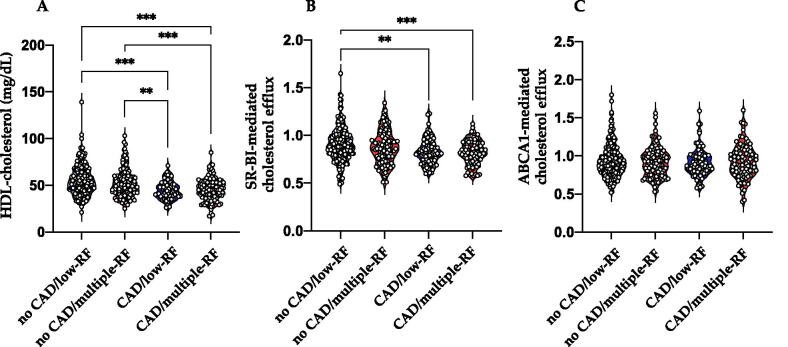
HDL-C levels and cholesterol efflux in patient subgroups. Patients from the CAPIRE study were stratified based on the number of risk factors (low-RF and multiple-RF) and the presence or absence of CAD (CAD and no-CAD). HDL-C levels (A), SR-BI-mediated cholesterol efflux (B), and ABCA1-mediated cholesterol efflux (C) were evaluated in each subgroup. **p < .01; ***p < .001 (One-way ANOVA, Tuckey post-hoc). RF: risk factor; CAD: coronary artery disease; HDL-C: high-density lipoprotein cholesterol; SR-BI: scavenger receptor class B type I; ABCA1: ATP binding cassette transporter A1.

The study population consisted of 525 patients, 263 in the low-RF group and 262 in the multiple RF group. Table 1 shows the clinical characteristics and laboratory data of low-RF and multiple-RF groups stratified by the absence or presence of CCTA-detected CAD. Mean HDL-C levels were significantly lower in patients with CAD in both groups (45.0 ± 9.7 mg/dL for low-RF patients with CAD; 45.5 ± 12.1 mg/dL for multiple-RF patients with CAD) compared to no-CAD subjects (55.2 ± 16 mg/dL for low-RF and 52.5 ± 15 mg/dL for multiple-RF); however, CAD patients in low-RF and multiple-RF groups had comparable levels of HDL-C (Figure 1A).

**Table 1.** Baseline characteristics by risk factor (RF) number and coronary artery disease (CAD) categories.

	Low-RF (*n* = 263)			Multiple-RF (*n* = 262)		
	No-CAD (*n* = 200)	CAD (SI*S* > 5) (*n* = 63)	*p*-value	No-CAD (*n* = 147)	CAD (SI*S* > 5) (*n* = 115)	*p*-value
*Demographic*						
Age, mean (SD), yrs	57.6 ± 8.6	63.6 ± 7.6	<.0001	57.8 ± 8.1	62.9 ± 6.9	<.0001
Male, %	49.5	93.7	<.0001	42.2	73.9	<.0001
BMI, mean (SD), Kg/m^2^	25.1 ± 3.7	26.8 ± 4.1	.0031	27.1 ± 4.0	28.3 ± 4.5	.025
*Medical history*						
Family History of IHD, %	7.0	6.4	.86	59.2	52.2	.26
Arterial hypertension, %	21.0	31.8	.09	77.6	86.9	.06
Dyslipidemia, %	48	43	.48	97.3	95.7	.47
Current smoking, %	4.5	6.4	.56	42.9	51.3	.17
Diabetes, %	–	–	–	19.7	31.3	.031
Systolic BP mean (SD), mmHg	125.1 ± 13.5	128.3 ± 13.4	.13	128.1 ± 14.7	135.9 ± 17.3	.004
Statin therapy, %	9	12.3	.43	46.3	59.1	.038
*Laboratory data*						
Total cholesterol, mean (SD), mg/dL	195.2 ± 39.3	188.0 ± 31.8	.14	210.5 ± 44.5	188.7 ± 44.5	.0001
LDL-cholesterol, mean (SD), mg/dL	119.3 ± 32.5	120.0 ± 28.9	.86	128.7 ± 34.9	118.3 ± 37.4	.0227
Triglycerides, median (IQR), mg/dL	73 (54–105)	104 (72–142)	<.0001	111 (76–178)	115 (84–165)	.62
HDL-cholesterol, mean (SD), mg/dL	55.2 ± 16.0	45.0 ± 9.7	<.0001	52.5 ± 15.1	45.5 ± 12.1	<.0001
SR-BI-mediated cholesterol efflux	0.90 ± 0.18	0.84 ± 0.14	.008	0.88 ± 0.17	0.82 ± 0.12	.002
ABCA1-mediated cholesterol efflux	0.93 ± 0.22	0.91 ± 0.19	.48	0.92 ± 0.18	0.92 ± 0.21	.97
C-reactive protein, median (IQR), mg/L	1.2 (0.5–2.7)	1.5 (0.5–4.6)	.194	1.7 (0.8–4.4)	2.4 (0.9–5.8)	.138
Serum creatinine, mean (SD), mg/dL	0.80 ± 0.16	0.91 ± 0.2	.0003	0.80 ± 0.18	0.87 ± 0.18	.004

RF: risk factor; CAD: coronary artery disease; SIS: segment involvement score; BMI: body mass index; IHD: ischaemic heart disease; BP: blood pressure; LDL: low-density lipoprotein; HDL: high-density lipoprotein; SR-BI: scavenger receptor class B type I; ABCA1: ATP binding cassette transporter A1; IQR: interquartile range; SD: standard deviation.

CEC was evaluated in all subgroups; SR-BI-mediated cholesterol efflux was significantly reduced in patients with CAD in both the low-RF and multiple-RF groups, whereas ABCA1-mediated cholesterol efflux was similar among all groups (Figure 1B and 1C). Since statins have been suggested to affect cholesterol efflux [29–31], we tested whether statin therapy might affect CEC in our study; we did not observe any significant differences in either SR-BI- or ABCA1-mediated cholesterol efflux between patients taking statins and patients not taking statin therapy (data not shown).

Considering HDL cholesterol efflux as a categorical variable, the prevalence of patients with diffuse CAD was significantly lower in patients in the highest quartile of SR-BI-mediated cholesterol efflux distribution (CEC > 75^th^ percentile vs ≤75^th^ OR: 0.39; 0.24–0.63; *p* < .0001); the prevalence was even lower when SR-BI-mediated cholesterol efflux >75^th^ percentile was combined with HDL-C >50mg/dL (OR 0.24; 0.13–0.44; *p* < .0001). Notably, the multivariable analysis applied in the two different risk groups showed that SR-BI-mediated cholesterol efflux >75^th^ percentile was associated with a lower prevalence of CAD, independently of HDL-C, in the multiple-RF group but not in the low-RF group (Supplementary Figure 1).

We then evaluated the correlation of SR-BI-mediated cholesterol efflux capacity with other continuous variables either in low-RF and multiple-RF subgroups, or no-CAD and CAD subgroups (Table 2A). Overall, SR-BI-mediated cholesterol efflux capacity showed significant positive correlations with TC and HDL-C levels, and significant inverse correlations with BMI, systolic BP, TG, CRP, and serum creatinine. When patients were stratified based on the number of RFs, most of these correlations were still significant both in low-RF and multiple-RF subgroups, while others were significant only in one of the two groups (Table 2A); similar findings were observed when patients were stratified based on the absence or presence of CAD (Table 2A). These correlations were less robust for ABCA-1-mediated cholesterol efflux (Table 2B).

**Table 2.** Correlation between SR-BI (A) or ABCA-1 (B) cholesterol efflux capacity and the main continuous variables.

	Overall		Low-RF		Multiple-RF		No-CAD		CAD		CAD/LRF		No CAD/MRF	
A	Rho	*p* value	Rho	*p* value	Rho	*p* value	Rho	*p* value	Rho	*p* value	Rho	*p* value	Rho	*p* value
Age, yrs	0.0042	.9231	0.0644	.2980	−0.0499	.4214	0.1163	.0303	−0.0414	.5832	−0.0109	.9326	0.0541	.5149
BMI, kg/m^2^	−**0.2376**	**<.0001**	**−0.2691**	**<.0001**	**−0.1509**	**.0145**	**−0.2367**	**<.0001**	−0.0958	.2032	−0.2320	.0673	**−0.1949**	**.0180**
Systolic BP, mmHg	**−0.1530**	**.0004**	−0.0986	.1105	**−0.1836**	**.0029**	−0.0886	.0996	**−0.1531**	**.0414**	−0.1650	.1963	−0.1262	.1277
Total cholesterol, mg/dL	**0.1228**	**.0048**	0.1149	.0629	**0.1472**	**.0171**	0.0634	.2391	**0.2005**	**.0073**	0.1384	.2795	−0.0092	.9123
LDL-cholesterol, mg/dL	0.0445	.3085	−0.0031	.9606	0.1052	.0893	−0.0348	.5180	**0.1774**	**.0178**	**0.4386**	**.0003**	**0.5382**	**<.0001**
Triglycerides, mg/dL	**−0.2472**	**<.0001**	**−0.2505**	**<.0001**	**−0.1926**	**.0017**	**−0.2443**	**<.0001**	−0.1462	.0515	0.0521	.6851	−0.0504	.5445
HDL-cholesterol, mg/dL	**0.4927**	**<.0001**	**0.4603**	**<.0001**	**0.5214**	**<.0001**	**0.4872**	**<.0001**	**0.4430**	**<.0001**	−0.2364	.0621	**−0.2687**	**.0010**
C-reactive protein, mg/L	**−0.1129**	**.0096**	**−0.1217**	**.0486**	−0.0802	.1957	−0.1032	.0548	−0.0568	.4513	−0.2308	.0687	−0.1551	.0607
IL-6, pg/ml	**−0.1169**	**.0073**	**−0.1858**	**.0025**	−0.0353	.5696	−0.1013	.0594	−0.1267	.0919	**−0.2729**	**.0305**	−0.0326	.6946
Serum creatinine, mg/dL	**−0.2082**	**<.0001**	**−0.1841**	**.0027**	**−0.2399**	**<.0001**	**−0.1699**	**.0015**	**−0.1826**	**.0147**	−0.1503	.2395	**−0.1924**	**.0195**

	Overall		Low RF		Multiple RF		No CAD		CAD		CAD/LRF		No CAD/MRF	
B	Rho	*p* value	*p* value	*p* value	*p* value	*p* value	*p* value	*p* value	*p* value	*p* value	*p* value	*p* value	*p* value	*p* value
Age, yrs	0.0310	.4785	.1345	.0293	−.0875	.1577	.0863	.1085	−.0423	.5754	.1322	.3017	−.0270	.7453
BMI, kg/m^2^	−0.0371	.3960	−.0268	.6656	−.0538	.3854	−.0519	.3355	.0014	.9853	.1578	.2167	−.0314	.7060
Systolic BP, mmHg	0.0050	.9088	.0481	.4370	−.0307	.6209	−.0268	.6182	.0803	.2869	.1258	.3260	−.1081	.1925
Total cholesterol, mg/dL	**0.3338**	**<.0001**	**.3109**	**<.0001**	**.3517**	**<.0001**	**.3695**	**<.0001**	**.2706**	**.0003**	.1787	.1610	**.4037**	**<.0001**
LDL-cholesterol, mg/dL	**0.2470**	**<.0001**	**.2091**	**.0006**	**.2705**	**<.0001**	**.2621**	**<.0001**	**.2174**	**.0036**	.1224	.3393	**.2877**	**.0004**
Triglycerides, mg/dL	0.0858	.0495	**.1482**	**.0161**	.0396	.5228	.0874	.1041	.0739	.3268	.1808	.1561	.0432	.6032
HDL-cholesterol, mg/dL	**0.1963**	**<.0001**	**.2124**	**.0005**	**.1890**	**.0021**	**.2330**	**<.0001**	.1468	.0505	.1302	.3092	**.2173**	**.0082**
C-reactive protein, mg/L	0.0039	.9290	−.0330	.5947	.0331	.5942	−.0222	.6796	.0611	.4180	−.0021	.9869	−.0126	.8796
IL-6, pg/ml	**0.1300**	**.0028**	.1099	.0752	**.1400**	**.0234**	**.1550**	**.0038**	.0879	.2431	.1033	.4204	**.1967**	**.0170**
Serum creatinine, mg/dL	**−0.1261**	**.0038**	**−.1296**	**.0357**	−.1201	.0522	**−.1379**	**.0101**	−.0997	.1854	−.1130	.3780	−.1394	.0922

Values (Rho) are Spearman’s correlation coefficients. RF: risk factor; LRF: low risk factors; MRF: multiple risk factors; BMI: body mass index; BP: blood pressure; LDL: low-density lipoprotein; HDL: high-density lipoprotein; IL-6: interleukin-6. Bold values denote statistical significance at the *p* < 0.05 level.

**Figure 2.**
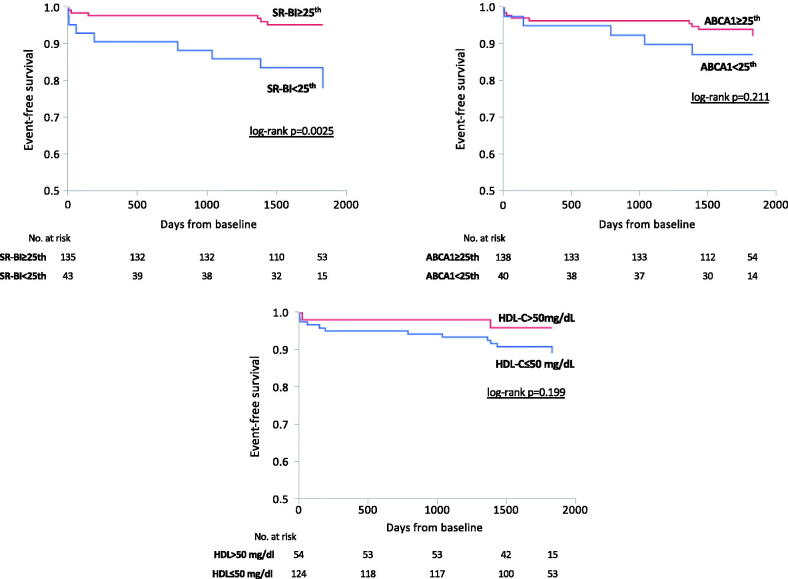
Kaplan-Meier event-free survival curves for death and acute coronary syndrome (ACS) stratified by SR-BI-mediated cholesterol efflux capacity, ABCA1-mediated cholesterol efflux capacity, and HDL-C levels. HDL-C: high density- lipoprotein cholesterol; SR-BI: scavenger receptor class B type I; ABCA1: ATP binding cassette transporter A1.

**Figure 3.**
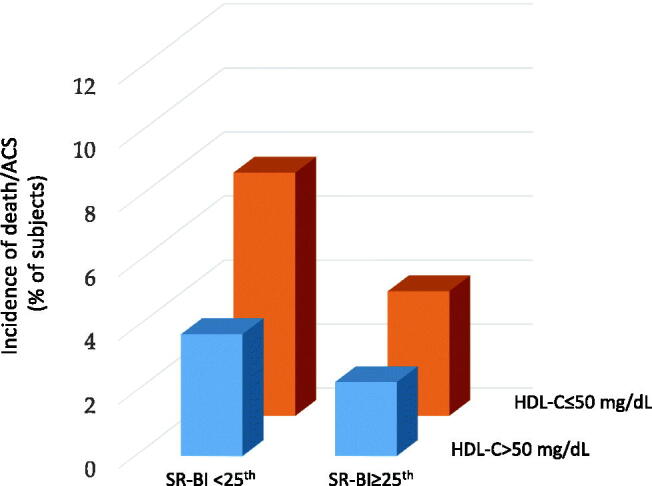
Prevalence of death ad ACS according to the interaction between HDL-C and SR-BI-mediated cholesterol efflux capacity. HDL-C: high-density lipoprotein cholesterol; SR-BI: scavenger receptor class B type I; ABCA1: ATP binding cassette transporter A1.

We next compared the association of HDL-C levels or cholesterol efflux with hard clinical events (death + ACS) during the 5-year follow-up. In patients with diffuse CAD, the reduction in SR-BI-mediated cholesterol efflux was significantly associated with an increase in clinical events (CEC <25^th^: 18.6%; 50–75^th^: 4.4% and >75^th^: 4.44%; *p* .011; CEC <25^th^ vs ≥25^th^ HR: 4.4, 1.54–13.4; log-rank *p* = .0026) (Figure 2). No changes were observed across ABCA1-mediated cholesterol efflux percentiles or HDL-C levels (Figure 2). Of note, a significant increase in the incidence of death + ACS was observed in patients having both SR-BI-mediated cholesterol efflux capacity <25^th^ percentile and HDL-C ≤50 mg/dL (Figure 3).

The association between SR-BI-mediated cholesterol efflux and atherosclerotic plaque characteristics identified by CCTA, including qualitative high-risk plaque features (PRI, LAP, NRS, and SC) or the presence of elevated total, non-calcified, and low-attenuation plaque volume was then assessed. SR-BI-mediated cholesterol efflux did not significantly associate with high-risk plaque features or the prevalence of elevated total, non-calcified, and low-attenuation plaque volume (Table 3). Similarly, a lack of association between ABCA-1-mediated cholesterol efflux or HDL-C levels and atherosclerotic plaque characteristics was also observed (Table 3).

**Table 3.** Baseline CCTA characteristics according to cholesterol efflux capacity distribution and HDL-C levels.

	SR-BI cholesterol efflux capacity				ABCA1 cholesterol efflux capacity				HDL-cholesterol		
	<25^th^ (n 43)	25–75^th^ (n 90)	>75^th^ (n 45)	*p* value	<25^th^ (n 40)	25–75^th^ (n 90)	>75^th^ (n 48)	*p* value	≤ 50 mg/dL (n 124)	> 50 mg/dL (n 54)	*p*value
CCTA characteristics											
Lumen stenosi*s* > 70%, *n* (%)	17 (39.5)	38 (42.2)	25 (55.6)	.243	18 (45)	46 (51.1)	16 (33.1)	.1354	57 (46)	23 (42.6)	.677
SSS, mean ± SD	11.6 ± 6.4	11.5 ± 6.0	11.9 ± 5.1	.93	12.3 ± 7.1	11.9 ± 5.8	10.5 ± 4.7	.273	11.8 ± 6.0	11.2 ± 5.4	.504
SIS, mean ± SD	7.6 ± 2.1	7.6 ± 2.0	7.6 ± 1.8	.989	7.6 ± 2.0	7.7 ± 2.1	7.4 ± 1.6	.627	7.6 ± 1.9	7.7 ± 2.0	.826
Leaman CT-adapted score, mean ± SD	10.3 ± 4.0	10.8 ± 4.4	10.6 ± 3.9	.831	10.6 ± 4.6	10.8 ± 4.0	10.2 ± 4.2	.700	10.9 ± 4.1	10.0 ± 4.3	.211
R*I* > 1.4, *n* (%)	30 (69.8)	53 (58.9)	21 (46.7)	.089	21 (52.5)	57 (63.3)	26 (54.2)	.401	76 (61.3)	28 (51.9)	.240
LAP, *n* (%)	29 (67.4)	56 (62.2)	32 (71.1)	.569	25 (62.5)	62 (68.9)	30 (62.5)	.668	86 (69.3)	31 (57.4)	.123
NRS, *n* (%)	14 (32.6)	14 (23.5)	11 (24.4)	.076	7 (17.5)	26 (28.9)	6 (12.5)	.064	34 (27.4)	5 (9.3)	.007
SC, *n* (%)	19 (44.2)	30 (33.3)	19 (42.2)	.394	14 (35.0)	33 (36.7)	21 (43.7)	.641	47 (37.9)	21 (38.9)	.901
More than 2 HRF per type, *n* (%)	13 (30.2)	20 (22.2)	15 (33.3)	.335	7 (17.5)	29 (32.2)	12 (25.0)	.204	36 (29.0)	12 (22.2)	.347
Pl Total Vol (mm^3^), median (IQR)	204 (100–312)	171 (103–277)	170 (100–277)	.823	174 (93–290)	180 (109–355)	162.5 (100–260)	.510	180 (102–312)	163.5 (101.5–266)	.328
Pl Vol H*U* < 150 (mm^3^), median (IQR)	45 (20–97)	31.5 (5–70)	29 (10.5–71.5)	.261	27 (5–84)	40.5 (15–86)	29 (7.5–70)	.346	40 (12.5–85.5)	25.5 (3.9–64.4)	.066
Pl Vol H*U* < 30 (mm^3^), median (IQR)	3 (0–17)	2 (0–7.4)	2.6 (0–9.5)	.481	2 (0–10)	3 (0–11)	2 (0–5)	.470	3 (0–10)	1 (0–5)	.086
Plaque length (mm), median (IQR)	46 (27–63)	40.5 (27.8–56)	46 (27.5–61)	.500	48 (32–63)	45.5 (28.7–63)	38.7 (24–50)	.041	43.5 (27–62)	39.5 (28–53)	.306
Pl Vol H*U* < 150/Plaque length	28.4 (12.8–39.8)	17.5 (6.6–30.2)	20.3 (9.0–31.9)	.051	14.7 (4.8–35.1)	22.4 (12.3–30.3)	14.3 (7.2–37.9)	.457	22.7 (11.1–37.2)	15 (6.6–27.4)	.062
Pl Vol H*U* < 30/Plaque length	0.10 (0–0.25)	0.05 (0–0.2)	0.04 (0–0.2)	.51	0.03 (0–0.23)	0.06 (0–0.22)	0.05 (0–0.19)	.638	0.07 (0–0.22)	0.03 (0–0.15)	.130
Myocardial mass, mean ± SD	115.7 ± 26.6	119.1 ± 23.8	121.1 ± 31.0	.630	115.6 ± 23.6	117.8 ± 24.5	123.1 ± 31.6	.383	118.7 ± 25.7	119.0 ± 28.1	.938

CCTA: coronary computed tomography angiography; SR–BI: scavenger receptor class B type I; ABCA1: ATP binding cassette transporter A1; HDL: high-density lipoproteins; SSS: segment stenosis score; SIS: segment involvement score; RI: Remodelling index; LAP: low attenuation plaque; NRS: napkin ring sing; SC: spotty calcification; HRF: high risk features; Pl Vol: plaque volume; IQR: interquartile range; SD: standard deviation.

Finally, in patients with CAD, we compared the prognostic value of SR-BI-mediated cholesterol efflux with variables previously found to be independently associated with clinical events, such as non-calcified atherosclerotic plaque (NCP) volume >80 mm^3^ and Framingham risk score (FRS) >20% [32]. Multivariable analysis showed that NCP > 80 mm^3^ (HR 3.9; 1.3–11.8: *p* = .014) and SR-BI-mediated cholesterol efflux <25^th^ percentile (HR 3.7; 1.3–11.3; *p* = .017) were significantly and independently associated with death + ACS outcome.

## Discussion

Although HDL-C levels and HDL function have been proposed as critical markers for improving CAD stratification [14–16], pharmacological strategies that substantially increased HDL-C levels failed to show any protective effect in large randomised clinical trials [33–36]. In addition, the results of Mendelian randomisation studies do not support a causal relationship between genetically determined HDL-C levels and the risk of cardiovascular events [8,37–40]. Several studies have thus explored the hypothesis that HDL function, rather than HDL-C levels, can be a relevant factor, with special attention to CEC [14–16], suggesting that improving HDL function might represent a valuable approach to reducing CV risk. In agreement with this assumption, a recent meta-analysis of 20 studies with a total of 25,132 subjects reported that higher CEC was associated with reduced incidence of CV outcomes, with the highest CEC group showing a 37% reduced risk of adverse CV events and 34% reduced risk of ASCVD [41]. Despite these observations, it is still unclear whether increasing CEC by pharmacological interventions might reduce the incidence of CV events, or even whether CEC evaluation might have prognostic usefulness.

In this cross-sectional analysis from the CAPIRE study, we specifically assessed the prognostic value of two indicators of HDL function, i.e. SR-BI- and ABCA-1-mediated cholesterol efflux, and found that higher SR-BI-mediated cholesterol efflux capacity, similarly to higher HDL-C levels, was associated with decreased CAD, in particular in patients with multiple risk factors, whereas lower SR-BI-mediated cholesterol efflux capacity was associated with worst clinical outcomes in patients with CAD, independently of atherosclerotic plaque features. Vice versa, the evaluation of ABCA-1-mediated cholesterol efflux neither improved patient stratification beyond traditional risk factors nor predicted an increased risk of cardiovascular events.

Overall, our analysis confirmed that SR-BI-mediated CEC mirrors the correlations between HDL-C levels and other cardiovascular risk factors, including a negative correlation with key markers of metabolic syndrome (BMI, systolic blood pressure, plasma triglyceride levels), markers of inflammation (CRP or IL-6), and a marker of kidney function. The latter observation extends previous findings in the general population [18,19], highlighting a critical role for improved HDL function in dampening kidney deterioration even in patients recruited in the CAPIRE study. Of note, the analysis of subgroups in the meta-analysis by Lee *et al* [41] reported that the inverse association between CEC and atherosclerotic CVD risk was observed among subjects without cardiovascular risk factors or chronic kidney disease (CKD) and individuals with cardiovascular risk factors, but not in patients with CKD. This finding might be related to the high heterogeneity of CKD patients included in the meta-analysis, and calls for more specifically designed studies to establish whether the use of CEC might improve risk prediction in this category of patients.

Several observations have suggested a potential involvement of statin therapy in improving HDL quality, and thus HDL function, or modulating genes encoding proteins participating in the reverse cholesterol transport [29–31]. Notably, in this study, we could not observe any significant effect of statin therapy on either SR-BI- or ABCA1-mediated CEC.

When patients from the CAPIRE study were stratified based on the number of RFs, SR-BI-mediated cholesterol efflux capacity correlated better with inflammatory markers in the low-RF cohort, whereas in the multiple-RF cohort it mainly correlated with markers of metabolic disorders. The inverse correlation between SB-RI-mediated cholesterol efflux and inflammatory markers in the low-RF cohort is in agreement with the hypothesis that HDL might control the immune-inflammatory response by modulating cholesterol content in immune cells [9,42,43].

In low-RF patients, the multivariable analysis did not show any evidence of superiority in using SR-BI- or ABCA1-mediated cholesterol efflux beyond that of HDL-C in identifying patients with CAD. Conversely, in the group of multiple-RF patients, the multivariable analysis showed that SR-BI-mediated cholesterol efflux largely improved the ability of HDL-C to discriminate patients without CAD, whereas ABCA-1-mediated cholesterol efflux did not.

Can this finding be explained by the correlation between SR-BI-mediated cholesterol efflux and atherosclerotic plaque features? We have previously shown that quantitative parameters of CCTA plaque assessment, more specifically the coronary plaque volume, and particularly the non-calcified plaque volume, are the most powerful predictors of cardiovascular events at follow-up, even beyond lumen stenosis and clinical risk profile [32]. Therefore, we tested the correlation between SR-BI- and ABCA-1-mediated cholesterol efflux and coronary plaque features, on the premise that improved cholesterol efflux should reduce cholesterol burden in the arteries and thus improve plaque features. Unexpectedly, total plaque volume was only slightly reduced in patients in the highest quartile of SR-BI-mediated cholesterol efflux. Furthermore, the prevalence of patients with severe lumen stenosis, arterial remodelling, plaque burden, napkin ring sign, or spotty calcification did not differ among quartiles of SR-BI-mediated cholesterol efflux. Similar observations were reported for other markers of atherosclerotic plaque quality and burden. These observations suggest that, although HDL function may be a predictor of cardiovascular disease, this role does not appear to be related to improved atherosclerotic plaque characteristics.

These observations are in contrast with findings from other studies. An inverse relationship between CEC and proteins associated with non-calcified plaque burden (that may represent a reversible stage in the atherosclerotic process and can regress following HDL infusion) was reported in adults with a clinical indication for a CCTA [44]; a similar inverse relationship between CEC and non-calcified burden plaque indices was observed among subjects with psoriasis, a chronic inflammatory skin disease associated with accelerated atherogenesis [45]. On the other hand, the CODAM study could not find any association between CEC and subclinical or clinical atherosclerosis [46].

We must acknowledge some limitations in our study; patients might have modified medication use or lifestyle after enrolment, which might have influenced the results. Moreover, the survival analysis included in this study was based on a relatively low number of cardiac events that occurred during the first 5 years of follow-up, and thus it should be considered of speculative interest. Another major challenge is the method used for CEC evaluation; this is not applicable in clinical practice, as it requires radiolabeled cholesterol and cultured cells; furthermore, any other assay for CEC evaluation may influence its association with cardiovascular outcomes. Of note, a simple, high-throughput, cell-free assay system has been established to assess the cholesterol uptake capacity (CUC) of HDL, which is a novel indicator for HDL functionality; the application of this method allowed to observe that CUC, but not HDL-C levels, was inversely associated with the lipid index and the macrophage score detected by optical coherence tomography [47].

In conclusion, SR-BI-mediated cholesterol efflux capacity is reduced in patients with diffuse coronary atherosclerosis and lower SR-BI-mediated cholesterol efflux capacity is associated with the worst clinical outcomes in patients with CAD, independently of atherosclerotic plaque features. Further studies are warranted to explore the mechanism(s) underlying these findings.

## Supplementary Material

Supplemental Material

## Data Availability

The data that support the findings of this study are available on request from the corresponding author.

## Appendix

### Steering committee

A. Maseri † (Chairman; Firenze), D. Andreini (Milano), S. Berti (Massa), M. Canestrari (Fano), G. Casolo (Lido di Camaiore), D. Gabrielli (Roma), R. Latini (Milano), M. Magnoni (Milano), P. Marraccini (Pisa), T. Moccetti (Lugano), M.G. Modena (Modena)

### Coordinating center

A.P. Maggioni, M. Gorini, F. Bianchini, I. Cangioli, A. Lorimer (Centro Studi ANMCO Fondazione per il Tuo cuore, Firenze)

### Imaging core laboratory

D. Andreini, G. Pontone, E. Conte (Centro Cardiologico Monzino Milano)

### Centralised biobank and biomarker core laboratory

D. Novelli, F. Gaspari, S. Ferrari, A. Cannata, N. Stucchi, M. Fois, R. Bernasconi, G. Balconi (Istituto Mario Negri, Milano and Bergamo), T. Vago, T. Letizia (Ospedale Luigi Sacco, Milano), B. Bottazzi, R. Leone (Istituto Clinico Humanitas, Rozzano)

### Central ECG reading

I. Suliman (Centro Studi ANMCO Fondazione per il Tuo cuore, Firenze)

### Psychologists CRF group

M. Sommaruga† (IRCCS Salvatore Maugeri, Unità di Psicologia, Milano), P. Gremigni (Dipartimento di Psicologia, Università di Bologna)

### Participating centers and investigators

Fano, Ospedale S Croce (R. Olivieri); Fermo, Ospedale Civile A. Murri (L. Pennacchietti); Lido di Camaiore, Nuovo Ospedale Versilia (M. Magnacca); Lugano, Cardiocentro Ticino (M.G. Rossi, E. Pasotti, T. Moccetti); Massa, FTGM – Stabilimento di Massa (A. Clemente); Milano, Centro Cardiologico Monzino (D. Andreini, G. Pontone, S. Mushtaq); Modena, Ospedale Policlinico (E. Mauro, G. Boriani); Parma, AOU. di Parma (F. Pigazzani); Pisa, AOU Pisana (L. Faggioni); Pisa, FTGM – Stabilimento di Pisa (M. Ciardetti); Udine, AOU SM della Misericordia (M. Puppato)
