# Supplementary material for: Predictive value of HDL function in patients with coronary artery disease: relationship with coronary plaque characteristics and clinical events
Source: Ann Med. 2022 Apr 19;54(1):1036–46. doi: 10.1080/07853890.2022.2063374 (PMC9090377; doi:10.1080/07853890.2022.2063374)
Supplement: Supplemental Material [file IANN_A_2063374_SM1499.pdf]

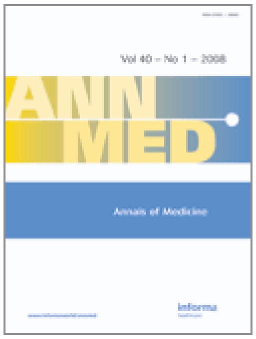

Predictive value of HDL function in patients with coronary artery disease: relationship with coronary plaque characteristics and clinical events

Journal:	*Annals of Medicine*
Manuscript ID	SANN-2022-OR-0116.R1
Manuscript Type:	Research Article
Date Submitted by the Author:	29-Mar-2022
Complete List of Authors:	Magnoni, Marco; Fondazione per il Tuo cuore, Heart Care Foundation Onlus<br>Andreini, Daniele; Centro Cardiologico Monzino Istituto di Ricovero e Cura a Carattere Scientifico, Department of Biomedical and Clinical Sciences<br>Pirillo, Angela; Ospedale Bassini, Center for the Study of Atherosclerosis<br>Uboldi, Patrizia; Universita degli Studi di Milano Biblioteca di Scienze del Farmaco, DiSFeB<br>Latini, Roberto; Istituto di Ricerche Farmacologiche Mario Negri, Department of Cardiovascular Medicine<br>Catapano, Alberico Luigi; University of Milan, DiSFeB and IRCCS Multimedica<br>Maggioni, Aldo Pietro; Associazione Nazionale Medici Cardiologi Ospedalieri, ANMCO<br>Norata, Giuseppe Danilo; Universita degli Studi di Milano Biblioteca di Scienze del Farmaco, DiSFeB
Keywords:	cholesterol efflux capacity, SR-BI, atherosclerotic plaque volume, coronary artery disease
Classifications:	

SCHOLARONE™
Manuscripts

Predictive value of HDL function in patients with coronary artery disease: relationship with coronary plaque characteristics and clinical events

Marco Magnoni[1], Daniele Andreini[2,3], Angela Pirillo[4,5], Patrizia Uboldi[6], Roberto Latini[7], Alberico L. Catapano[5,6], Aldo P. Maggioni[8], Giuseppe D. Norata[5,6], on behalf of the CAPIRE Study Group

Running head: Cholesterol efflux and coronary plaque features

1. Heart Care Foundation Onlus, Florence, Italy.
2. Centro Cardiologico Monzino, IRCCS, Milan, Italy.
3. Department of Biomedical and Clinical Sciences "Luigi Sacco", University of Milan, Milan, Italy
4. Centro SISA per lo Studio dell'Aterosclerosi, Ospedale Bassini, Cinisello Balsamo, Italy.
5. IRCSS Multimedica, Milan, Italy.
6. Department of Excellence of Pharmacological and Biomolecular Sciences, Università degli Studi di Milano, Milan, Italy.
7. Department of Cardiovascular Medicine, IRCCS - Istituto di Ricerche Farmacologiche "Mario Negri", Milan, Italy
8. ANMCO Research Center, Florence, Italy

For Peer Review Only

Abstract

Background. HDL are endowed with several metabolic, vascular, and immunoinflammatory-protective functions. Among them, a key property is to promote reverse cholesterol transport from cells back to the liver.

The aim of this study was to estimate the association of scavenger receptor class B type I (SR-BI)- and ATP binding cassette transporter A1 (ABCA-1)-mediated cholesterol efflux (the two major routes for cholesterol efflux to HDL) with the presence, extent, and severity of coronary artery disease (CAD), vascular wall remodeling processes, coronary plaque characteristics, and the incidence of myocardial infarction in the different subgroups of patients from the CAPIRE study.

Methods. Patients (n=525) from the CAPIRE study were divided into two groups: low-risk factors (RF), with 0-1 RF (n=263), and multiple-RF, with ≥2 RFs; within each group, subjects were classified as no-CAD or CAD based on the segment involvement score (SIS) evaluated by coronary computed tomography angiography (SIS=0 and SIS>5, respectively). SR-BI- and ABCA1-mediated cholesterol efflux were measured using the plasma of all patients.

Results. SR-BI-mediated cholesterol efflux was significantly reduced in patients with CAD in both the low-RF and multiple-RF groups, whereas ABCA1-mediated cholesterol efflux was similar among all groups. In CAD patients, multivariable analysis showed that SR-BI-mediated cholesterol efflux <25th percentile predicted cardiovascular outcome (odds ratio 4.1; 95% CI: 1.3-13.7; p=0.019), whereas ABCA-1-mediated cholesterol efflux and HDL-C levels significantly did not. In spite of this finding, reduced SR-BI-mediated cholesterol efflux was not associated with changes in high-risk plaque features or changes in the prevalence of elevated total, non-calcified, and low-attenuation plaque volume.

Conclusion. SR-BI-mediated cholesterol efflux capacity is lower in patients with diffuse coronary atherosclerosis. In addition, a lower SR-BI-mediated cholesterol efflux capacity is associated with worst clinical outcomes in patients with CAD, independently of atherosclerotic plaque features.

Keywords: Cholesterol efflux capacity; SR-BI; atherosclerotic plaque volume; coronary artery disease

Email: IANN-peerreview@journals.tandf.co.uk URL: http://mc.manuscriptcentral.com/sann

Key Messages

- Increased cholesterol efflux capacity, an estimate of HDL function, is associated with a reduced CVD risk, regardless of HDL-C levels
- HDL-C levels are significantly lower in patients with CAD
- Lower SR-BI-mediated cholesterol efflux capacity is observed in patients with diffuse coronary atherosclerosis and is associated with worst clinical outcomes in patients with CAD, independently of atherosclerotic plaque features

For Peer Review Only

Introduction

Coronary artery disease (CAD) is a leading cause of death, despite continuous improvements in prevention and treatment [1]. The risk of coronary events is conventionally calculated by multifactorial stratification methods that integrate traditional risk factors (RFs) [2, 3]. Among different RFs, epidemiological data highlighted an inverse relationship between high-density lipoprotein cholesterol (HDL-C) levels and the incidence of cardiovascular disease (CVD), at least for values up to 80-90 mg/dL [4, 5], while at higher levels a U-shaped association has been demonstrated, with extremely high HDL-C levels being associated with an increased CVD risk [6]. Uncertainty on the causal role of HDL in CVD is still elevated [6-8]; a possible explanation is that HDL-C levels do not always provide information on the functionality of HDL particles in a specific setting.

Indeed, HDL are endowed with several metabolic, vascular, and immunoinflammatory-protective functions [9, 10]. Among them, a key property is to promote reverse cholesterol transport, i.e. cholesterol efflux from cells back to the liver [11]. This evolutionarily conserved mechanism initiates with the ATP binding cassette transporter A1 (ABCA1)-mediated unidirectional export of cholesterol and phospholipids from cells to lipid-poor apolipoprotein A-I (the main apolipoprotein of HDL), leading to the formation of nascent, discoidal pre-ß-HDL particles. Next, two transporters, namely scavenger receptor class B type I (SR-BI, which mediates a bidirectional flux of free cholesterol between cells and HDL) and ABCG1 mediate further cholesterol efflux and contribute to HDL maturation and the generation of large, spherical HDL particles. The assessment of cholesterol efflux capacity (CEC) has been adopted as an estimate of HDL function [11]; of note, HDL particles exhibit high levels of structural and compositional heterogeneity, and phospholipid content and composition are major factors determining the HDL cholesterol efflux capacity [12, 13]. Several studies have investigated the association between CEC and the incidence of cardiovascular events in the general population, and many, but not all, observed that an increased CEC is associated with a reduced CVD risk, regardless of HDL-C concentration [14-17]. This association, however, is not as robust in patients with reduced kidney function [18] or end-stage renal disease [19] in whom, on the other hand, HDL-C levels predict

Email: IANN-peerreview@journals.tandf.co.uk URL: http://mc.manuscriptcentral.com/sann

For Peer Review Only

disease progression [18, 19], suggesting that HDL function can be a good predictor of CVD, at least in initially healthy individuals without clinically manifest CVD.

Early discrimination of subjects with established CVD but no clinical symptoms is an emerging area of discussion where the possibility to strengthen traditional RF-based CV risk assessment by including a direct estimation of coronary atherosclerosis represents an intriguing option. Coronary computed tomography angiography (CCTA) is a comprehensive non-invasive diagnostic test that provides information about the presence, extent, and severity of CAD, vascular wall remodeling processes, and plaque characteristics. It enables to identify subjects with normal coronary arteries (high negative predictive value) or with subclinical disease, and it better defines the global atherosclerotic process [20]. Furthermore, CCTA allows identifying patients with unexpected diffuse CAD despite a low RF profile, as well as those who, despite the presence of multiple RFs, develop only mild or no coronary atherosclerosis [21-23]. These extreme "outlier" populations have been specifically investigated in the CAPIRE (Coronary Atherosclerosis in outlier subjects: Protective and novel Individual Risk factor Evaluation) study [24], a hypothesis-generating study aimed at exploring protective and susceptibility factors of CAD to identify high-risk subjects who may benefit from a more personalized prevention strategy.

The aims of this study were therefore to estimate HDL CEC, discriminating between cholesterol efflux to nascent and mature HDL, and its association with coronary artery characteristics and the incidence of myocardial infarction (MI) in the population from the CAPIRE study.

For Peer Review Only

METHODS

CAPIRE study

The CAPIRE (Coronary Atherosclerosis in Outlier Subjects: Protective and Individual Risk Factor Evaluation) study (NCT02157662) is part of the GISSI Outlier Project; it is a prospective, observational, international multicenter study involving a cross-sectional comparison of several variables (clinical, imaging, and biomolecular) with a 10-year follow-up [24]; the data presented here refer to a 5-year follow-up period.

Study population

For this study, 525 consecutive patients aged 45 to 75 years without acute coronary syndrome and with normal left ventricular ejection fraction were recruited. Participants underwent 64-slice (or superior) CCTA for suspected CAD in the outpatient clinics of the 11 centers involved in the study, and, based on CCTA result and risk profile, they were divided into four groups, according to pre-specified criteria:

- Low-RF/no-CAD: subjects with 0-1 RF (with the exclusion of patients with type 1 or type 2 diabetes mellitus as single RF) and no CAD;
- Low-RF/CAD: subjects with 0-1 RF (with the exclusion of patients with type 1 or type 2 diabetes mellitus as single RF) and diffuse CAD extended to >5 of the 16 segments defined by the American Heart Association (AHA) classification [25];
- Multiple-RF/no-CAD/: subjects with ≥ 2 RFs and no CAD;
- Multiple-RF/CAD: subjects with ≥2 RFs and diffuse CAD extended to >5 segments.

Patients with low CCTA quality control criteria, or reporting previous cardiovascular events (including acute MI, unstable or chronic stable angina, percutaneous or surgical coronary revascularization, and heart failure), dilated cardiomyopathy, obstructive hypertrophic cardiomyopathy, atrial fibrillation, myocarditis, inflammatory vascular disease, acute or chronic peripheral vascular disease, active inflammatory, or neoplastic disease were not enrolled in the study. The protocol was approved by the local Ethical Committee of each participant site and all patients provided informed consent. The list of participating centers is provided in the Appendix section.

For Peer Review Only

Risk factors included family history of CAD (history of early manifestations of CAD in first-degree relatives, <55 years old for men and <65 years old for women), systemic hypertension (history of arterial hypertension, ongoing antihypertensive treatment, or recent observation of blood pressure values >140/90 mmHg), hypercholesterolemia (total cholesterol >200 mg/dl or <200 mg/dl if under lipid-lowering therapy), diabetes mellitus (fasting plasma blood glucose levels >126 mg/dL, or 2-hour values in the oral glucose tolerance test ≥200 mg/dL, or isolated elevation of glycated hemoglobin ≥6.5%, or current use of insulin or oral hypoglycemic agents), and cigarette smoking (current smoker or < 1-year abstention) [26]. Physical examination, anamnestic records, and laboratory tests provided by the participants or documented before CCTA were used to define individual's risk factors. Lipid profile and metabolic parameters were evaluated also in a centralized core laboratory to validate the local assessment of RFs such as diabetes or hypercholesterolemia.

Laboratory analysis

A peripheral venous blood sample was collected from each patient at the enrolment. The samples were immediately processed to obtain separate aliquots of whole blood, plasma, and serum and stored at -70°C in a dedicated biological bank (HCF blood bank, located at SATURNE-1, Mario Negri Institute of Pharmacological Research, Milan).

Circulating biomarkers were measured in a central laboratory, in a single batch, by personnel unaware of patients' characteristics. High-sensitivity C-reactive protein was measured with an automatic immunoturbidimetric method (Beckman-Coulter, Galway, Ireland). Serum creatinine and lipids were measured with standard, automated laboratory methods.

HDL-mediated cholesterol efflux evaluation

To evaluate SR-BI-mediated cholesterol efflux, Fu5AH cells were grown to subconfluence, then incubated for 24h with DMEM containing 5% FCS, ^{3}H-cholesterol (1 μCi/ml), and 2 μg/ml ACAT inhibitor Sandoz 58-035. After washing, cells were incubated overnight in fresh DMEM containing 0.2% BSA and 2 μg/ml Sandoz 58-035. For efflux, cells were incubated with 1.5% plasma diluted in serum-free medium for 4 hours. The media were

For Peer Review Only

collected, centrifuged, and aliquots were used for liquid scintillation counting. Cell monolayers were lysed with 0.1N NaOH and aliquots were used for liquid scintillation counting. The efflux of ^{3}H-cholesterol was calculated as the ratio of radioactivity released into the medium to the total (medium plus intracellular) radioactivity. To correct for inter-assay variation across plates, a pooled plasma control from two healthy volunteers was included on each plate, and values for plasma samples from patients were normalized to this pooled value in all analyses. Intra- and inter-assay coefficients of variation for SR-BI-mediated cholesterol efflux were 4.7% and 14.3%, respectively.

To investigate the role of ABCA1 in cholesterol efflux, J774 cells were labelled for 24h with MEM containing 50 μg/ml AcLDL, ^{3}H-cholesterol (1 μCi/ml), and 2 μg/ml Sandoz 58-035. After washing, cells were incubated for 18 hours in MEM containing 0.2% BSA, 0.3 mM 8-Br-cAMP, and 2 μg/ml Sandoz 58-035. For efflux, cells were incubated with 1% plasma of individual patients diluted in serum-free medium for 4 hours. Samples were processed as described above.

Coronary CTA Analysis

All CCTA scans were transferred to the CCTA Core Lab (Centro Cardiologico Monzino, Milano) for a central blinded analysis of coronary angiograms.

Coronary plaques were defined as structures of at least 1 mm^2 area within and/or adjacent to artery lumen, clearly distinguishable from the vessel lumen, and surrounded by pericardial tissue; tissue with signal intensity below -40HU was considered a pericardial fat and excluded from the analysis. Coronary arteries were referred to as normal in the absence of atherosclerotic plaque (including focal and eccentric calcified plaques) in each segment.

Details on the evaluation of high-risk plaque features (HPFs) have been reported previously [27]. Plaque consistency was evaluated using Hounsfield Unit (HU); low-attenuation plaque volume and non-calcified plaque volume were defined as <30 HU and <150 HU, respectively, and expressed in mm^3 [28]. Total plaque volume was evaluated and reported in mm^3. All plaque volumes have also been evaluated as qualitative dichotomous variables using the higher quartile as cut-off on a per-patient basis.

Email: IANN-peerreview@journals.tandf.co.uk URL: http://mc.manuscriptcentral.com/sann

For Peer Review Only

Five-years follow-up findings

Clinical visits were scheduled every 12 months, with structured phone interviews planned every 6 months. All clinical events were recorded and validated centrally by an event committee blinded to the CCTA results. Hospital records and outsourced clinical documents were screened to confirm the information obtained. For this analysis, data on cardiac death, acute coronary syndrome (ACS), and non-urgent revascularization were recorded. The definition of these events has been reported in detail in a previous paper [27].

Statistical analysis

Continuous variables were presented as mean±SD or median with interquartile range (25th -75th) (for non-normal distribution). Student's t-test for independent samples and the analysis of variance (ANOVA) for repeated measurements were used to compare continuous normally distributed variables; Mann-Whitney U tests for independent samples and Wilcoxon test for repeated measurements were used for non-normal distributions. The proportion of categorical variables was compared using a Chi-square analysis or Fisher's exact test. Values of $p<0.05$ were considered statistically significant. Odds ratios (ORs) with 95% confidence intervals (CIs) and estimated hazard ratios (HR) with 95% CIs were presented for each class of the variables that were significant in the univariate analysis. The event-free survival curves were analyzed using the Kaplan–Meier method and compared using the log-rank test. Cox regression analysis was performed to identify the independent predictors of clinical outcomes. The multivariable model was created including all the variables with a probability value of <0.05 in the univariate analysis.

Statistical analysis was performed using SAS (version 11, SAS Institute Inc. 2013 Cary, North Carolina) and JMP software (version 11.0.0, SAS Institute Inc., Cary, North Carolina, USA). Comparisons of areas under the ROC curve were performed using MedCalc Statistical Software (version 12.3.0, MedCalc Software bvba2013, Ostend, Belgium).

For Peer Review Only

RESULTS

The study population consisted of 525 patients, 263 in the low-RF group and 262 in the multiple RF group. Table 1 shows the clinical characteristics and laboratory data of low-RF and multiple-RF groups stratified by the absence or presence of CCTA-detected CAD. Mean HDL-C levels were significantly lower in patients with CAD in both groups (45.0±9.7 mg/dL for low-RF patients with CAD; 45.5±12.1mg/dL for multiple-RF patients with CAD) compared to no-CAD subjects (55.2±16 mg/dL for low-RF and 52.5±15 mg/dL for multiple-RF); however, CAD patients in low-RF and multiple-RF groups had comparable levels of HDL-C (Figure 1A).

CEC was evaluated in all subgroups; SR-BI-mediated cholesterol efflux was significantly reduced in patients with CAD in both the low-RF and multiple-RF groups, whereas ABCA1-mediated cholesterol efflux was similar among all groups (Figure 1B and 1C). Since statins have been suggested to affect cholesterol efflux [29-31], we tested whether statin therapy might affect CEC in our study; we did not observe any significant differences in either SR-BI- or ABCA1-mediated cholesterol efflux between patients taking statins and patients not taking statin therapy (data not shown).

Considering HDL cholesterol efflux as a categorical variable, the prevalence of patients with diffuse CAD was significantly lower in patients in the highest quartile of SR-BI-mediated cholesterol efflux distribution (CEC>75th percentile vs ≤75th OR: 0.39; 0.24-0.63; p<0.0001); the prevalence was even lower when SR-BI-mediated cholesterol efflux >75th percentile was combined with HDL >50mg/dL (OR 0.24; 0.13-0.44; p<0.0001). Notably, the multivariable analysis applied in the two different risk groups showed that SR-BI-mediated cholesterol efflux >75th percentile was associated with a lower prevalence of CAD, independently of HDL-C, in the multiple-RF group but not in the low-RF group (Supplementary Figure 1).

We then evaluated the correlation of SR-BI-mediated cholesterol efflux capacity with other continuous variables either in low-RF and multiple-RF subgroups, or no-CAD and CAD subgroups (Table 2A). Overall, SR-BI-mediated cholesterol efflux capacity showed significant positive correlations with TC and HDL-C levels, and significant inverse correlations with BMI, systolic BP, TG, CRP, and serum creatinine. When patients were

For Peer Review Only

stratified based on the number of RFs, most of these correlations were still significant both in low-RF and multiple-RF subgroups, while others were significant only in one of the two groups (Table 2A); similar findings were observed when patients were stratified based on the absence or presence of CAD (Table 2A). These correlations were less robust for ABCA-1-mediated cholesterol efflux (Table 2B).

We next compared the association of HDL-C levels or cholesterol efflux with hard clinical events (death+ACS) during the 5-year follow-up. In patients with diffuse CAD, the reduction in SR-BI-mediated cholesterol efflux was significantly associated with an increase in clinical events (CEC <25th: 18.6%; 50-75th: 4.4% and >75th: 4.44%; p 0.011; CEC <25th vs ≥25th HR: 4.4, 1.54-13.4; log-rank p=0.0026) (Figure 2). No changes were observed across ABCA1-mediated cholesterol efflux percentiles or HDL-C levels (Figure 2). Of note, a significant increase in the incidence of death+ACS was observed in patients having both SR-BI-mediated cholesterol efflux capacity <25th percentile and HDL ≤50 mg/dL (Figure 3).

The association between SR-BI-mediated cholesterol efflux and atherosclerotic plaque characteristics identified by CCTA, including qualitative high-risk plaque features (PRI, LAP, NRS, and SC) or the presence of elevated total, non-calcified, and low-attenuation plaque volume was then assessed. SR-BI-mediated cholesterol efflux did not significantly associate with high-risk plaque features or the prevalence of elevated total, non-calcified, and low-attenuation plaque volume (Table 3). Similarly, a lack of association between ABCA-1-mediated cholesterol efflux or HDL-C levels and atherosclerotic plaque characteristics was also observed (Table 3).

Finally, in patients with CAD, we compared the prognostic value of SR-BI-mediated cholesterol efflux with variables previously found to be independently associated with clinical events, such as non-calcified atherosclerotic plaque (NCP) volume >80 mm^3 and Framingham risk score (FRS) >20% [32]. Multivariable analysis showed that NCP>80 mm^3 (HR 3.9; 1.3-11.8: p=0.014) and SR-BI-mediated cholesterol efflux <25th percentile (HR 3.7; 1.3-11.3; p=0.017) were significantly and independently associated with death+ACS outcome.

For Peer Review Only

DISCUSSION

Although HDL-C levels and HDL function have been proposed as critical markers for improving CAD stratification [14-16], pharmacological strategies that substantially increased HDL-C levels failed to show any protective effect in large randomized clinical trials [33-36]. In addition, the results of Mendelian randomization studies do not support a causal relationship between genetically determined HDL-C levels and the risk of cardiovascular events [8, 37-40]. Several studies have thus explored the hypothesis that HDL function, rather than HDL-C levels, can be a relevant factor, with special attention to CEC [14-16], suggesting that improving HDL function might represent a valuable approach to reduce CV risk. In agreement with this assumption, a recent meta-analysis of 20 studies with a total of 25,132 subjects reported that higher CEC was associated with reduced incidence of CV outcomes, with the highest CEC group showing a 37% reduced risk of adverse CV events and 34% reduced risk of ASCVD [41]. Despite these observations, it is still unclear whether increasing CEC by pharmacological interventions might reduce the incidence of CV events, or even whether CEC evaluation might have prognostic usefulness.

In this cross-sectional analysis from the CAPIRE study, we specifically assessed the prognostic value of two indicators of HDL function, i.e. SR-BI- and ABCA-1-mediated cholesterol efflux,-and found that higher SR-BI-mediated cholesterol efflux capacity, similar to higher HDL-C levels, was associated with decreased CAD, in particular in patients with multiple risk factors, whereas lower SR-BI-mediated cholesterol efflux capacity was associated with worst clinical outcomes in patients with CAD, independently of atherosclerotic plaque features. Vice versa, the evaluation of ABCA-1-mediated cholesterol efflux neither improved patient stratification beyond traditional risk factors nor predicted an increased risk of cardiovascular events.

Overall, our analysis confirmed that SR-BI-mediated CEC mirrors the correlations of HDL-C levels with other cardiovascular risk factors, including a negative correlation with key markers of metabolic syndrome (BMI, systolic blood pressure, plasma triglyceride levels), markers of inflammation (CRP or IL-6), and a marker of kidney function. The latter observation extends previous findings in the general population [18, 19], highlighting a

Email: IANN-peerreview@journals.tandf.co.uk URL: http://mc.manuscriptcentral.com/sann

critical role for improved HDL function in dampening kidney deterioration even in patients recruited in the CAPIRE study. Of note, the analysis of subgroups in the meta-analysis by Lee et al [41] reported that the inverse association between CEC and atherosclerotic CVD risk was observed among subjects without cardiovascular risk factors or chronic kidney disease (CKD) and individuals with cardiovascular risk factors, but not in patients with CKD. This finding might be related to the high heterogeneity of CKD patients included in the meta-analysis, and calls for more specifically designed studies to establish whether the use of CEC might improve risk prediction in this category of patients.

Several observations have suggested a potential involvement of statin therapy in improving HDL quality, and thus HDL function, or modulating genes encoding proteins participating in the reverse cholesterol transport [29-31]. Notably, in this study, we could not observe any significant effect of statin therapy on either SR-BI- or ABCA1-mediated CEC.

When patients from the CAPIRE study were stratified based on the number of RFs, SR-BI-mediated cholesterol efflux capacity correlated better with inflammatory markers in the low-RF cohort, whereas in the multiple-RF cohort it mainly correlated with markers of metabolic disorders. The inverse correlation between SB-RI-mediated cholesterol efflux and inflammatory markers in the low-RF cohort is in agreement with the hypothesis that HDL might control the immune-inflammatory response by modulating cholesterol content in immune cells [9, 42, 43].

In low-RF patients, the multivariable analysis did not show any evidence of superiority in using SR-BI- or ABCA1-mediated cholesterol efflux beyond that of HDL-C in identifying patients with CAD. Conversely, in the group of multiple-RF patients, the multivariable analysis showed that SR-BI-mediated cholesterol efflux largely improved the ability of HDL-C to discriminate patients without CAD, whereas ABCA-1-mediated cholesterol efflux did not.

Can this finding be explained by the correlation between SR-BI-mediated cholesterol efflux and atherosclerotic plaque features? We have previously shown that quantitative parameters of CCTA plaque assessment, more specifically the coronary plaque volume, and particularly the non-calcified plaque volume, are the most powerful predictors of

Email: IANN-peerreview@journals.tandf.co.uk URL: http://mc.manuscriptcentral.com/sann

For Peer Review Only

cardiovascular events at follow-up, even beyond lumen stenosis and clinical risk profile [32]. Therefore, we tested the correlation between SR-BI- and ABCA-1-mediated cholesterol efflux and coronary plaque features, on the premise that improved cholesterol efflux should reduce cholesterol burden in the arteries and thus improve plaque features. Unexpectedly, total plaque volume was only slightly reduced in patients in the highest quartile of SR-BI-mediated cholesterol efflux. Furthermore, the prevalence of patients with severe lumen stenosis, arterial remodeling, plaque burden, napkin ring sign, or spotty calcification did not differ among quartiles of SR-BI-mediated cholesterol efflux. Similar observations were reported for other markers of atherosclerotic plaque quality and burden. These observations suggest that, although HDL function may be a predictor of cardiovascular disease, this role does not appear to be related to improved atherosclerotic plaque characteristics.

These observations are in contrast with findings from other studies. An inverse relationship between CEC and proteins associated with non-calcified plaque burden (that may represent a reversible stage in the atherosclerotic process and can regress following HDL infusion) was reported in adults with a clinical indication for a CCTA [44]; a similar inverse relationship between CEC and non-calcified burden plaque indices was observed among subjects with psoriasis, a chronic inflammatory skin disease associated with accelerated atherogenesis [45]. On the other hand, the CODAM study could not find any association between CEC and subclinical or clinical atherosclerosis [46].

We must acknowledge some limitations in our study; patients might have modified medication use or lifestyle after enrollment, which might have influenced the results. Moreover, the survival analysis included in this study was based on a relatively low number of cardiac events that occurred during the first 5 years of follow-up, and thus it should be considered of speculative interest. Another major challenge is the method used for CEC evaluation; this is not applicable in clinical practice, as it requires radiolabeled cholesterol and cultured cells; furthermore, any other assay for CEC evaluation may influence its association with cardiovascular outcomes. Of note, a simple, high-throughput, cell-free assay system has been established to assess the cholesterol uptake capacity (CUC) of HDL, which is a novel indicator for HDL functionality; the application of this method allowed to

For Peer Review Only

observe that CUC, but not HDL-C levels, was inversely associated with the lipid index and the macrophage score detected by optical coherence tomography [47].

In conclusion, SR-BI-mediated cholesterol efflux capacity is reduced in patients with diffuse coronary atherosclerosis and lower SR-BI-mediated cholesterol efflux capacity is associated with worst clinical outcomes in patients with CAD, independently of atherosclerotic plaque features. Further studies are warranted to explore the mechanism(s) underlying these findings.

For Peer Review Only

Figure Legends

Figure 1. HDL-C levels and cholesterol efflux in patient subgroups. Patients from the CAPIRE study were stratified based on the number of risk factors (low-RF and multiple-RF) and the presence or absence of CAD (CAD and no-CAD). HDL-C levels **(A)**, SR-BI-mediated cholesterol efflux **(B)**, and ABCA1-mediated cholesterol efflux **(C)** were evaluated in each subgroup.
**p<0.01; ***p<0.001 (One-way ANOVA, Tuckey post-Hoc)
RF: risk factor; CAD: coronary artery disease; HDL-C: high-density lipoprotein cholesterol; SR-BI: scavenger receptor class B type I; ABCA1: ATP binding cassette transporter A1

Figure 2. Kaplan-Meier event-free survival curves for death and acute coronary syndrome (ACS) stratified by SR-BI-mediated cholesterol efflux capacity, ABCA1-mediated cholesterol efflux capacity, and HDL-C levels.
HDL-C: high density- lipoprotein cholesterol; SR-BI: scavenger receptor class B type I; ABCA1: ATP binding cassette transporter A1
Figure 3. Prevalence of death ad ACS according to the interaction between HDL-C and SR-BI-mediated cholesterol efflux capacity.
HDL-C: high-density lipoprotein cholesterol; SR-BI: scavenger receptor class B type I; ABCA1: ATP binding cassette transporter A1

Supplementary Figure 1. Prevalence of patients with extensive CAD at CCTA according to HDL-C and SR-BI-mediated cholesterol efflux capacity in patients with low-RF or multiple-RF.
CAD: coronary artery disease; CCTA: coronary computed tomography angiography; HDL-C: high-density lipoprotein cholesterol; SR-BI: scavenger receptor class B type I; RF: risk factors

For Peer Review Only

Appendix

Steering Committee

A. Maseri † (Chairman; Firenze), D. Andreini (Milano), S. Berti (Massa), M. Canestrari (Fano), G. Casolo (Lido di Camaiore), D. Gabrielli (Roma), R. Latini (Milano), M. Magnoni (Milano), P. Marraccini (Pisa), T. Moccetti (Lugano), M.G. Modena (Modena)

Coordinating Center

A.P. Maggioni, M. Gorini, F. Bianchini, I. Cangioli, A. Lorimer (Centro Studi ANMCO Firenze)

Imaging Core Laboratory

D. Andreini, G. Pontone, E. Conte (Centro Cardiologico Monzino Milano)

Centralized biobank and biomarker core laboratory

D. Novelli, F. Gaspari, S. Ferrari, A. Cannata, N. Stucchi, M. Fois, R. Bernasconi, G. Balconi (Istituto Mario Negri, Milano and Bergamo), T. Vago, T. Letizia (Ospedale Luigi Sacco, Milano), B. Bottazzi, R. Leone (Istituto Clinico Humanitas, Rozzano).

Central ECG Reading

I. Suliman (Centro Studi ANMCO, Firenze)

Psychologists CRF Group

M. Sommaruga† (IRCCS Salvatore Maugeri Unità di Psicologia, Milano), P. Gremigni (Dipartimento di Psicologia Università di Bologna)

Participating Centers and Investigators

Fano, Ospedale S Croce (R. Olivieri); Fermo, Ospedale Civile A. Murri (L. Pennacchietti); Lido di Camaiore, Nuovo Ospedale Versilia (M. Magnacca); Lugano, Cardiocentro Ticino (M.G. Rossi, E. Pasotti, T. Moccetti); Massa, IFC-CNR Ospedale Pasquinucci, Cardiologia; Modena, Ospedale Policlinico (E. Mauro, G. Boriani); Parma, AOU. di Parma (F. Pigazzani); Pisa, AOU Pisana (L. Faggioni); Pisa, FTGM - Stabilimento di Pisa (M. Ciardetti); Udine, AOU SM della Misericordia (M. Puppato)

For Peer Review Only

Author Contributions statement

MM: contributed to design, data acquisition, analysis, and interpretation, and drafted the manuscript; AD: contributed to conception and critically revised the manuscript; AP: contributed to data interpretation and drafted the manuscript; UP: contributed to data acquisition and analysis; LR: contributed to study conception, critically revised the manuscript; ALC: Contributed to study conception and critically revised the manuscript. APM: contributed to design and data interpretation, and critically revised the manuscript; GDN: contributed to design, data acquisition, analysis, and interpretation, drafted the manuscript, and critically revised the manuscript.

Disclosure of interest

The authors have no conflicts of interest to disclose.

Data availability statement

The data that support the findings of this study are available on request from the corresponding author

Funding

Fundings were provided by the Heart Care Foundation of the Italian Association of Hospital Cardiologists, Florence, Italy, Telethon Foundation GGP19146 to GDN; PRIN 2017H5F943 to ALC, PRIN 2017K55HLC to GDN, Ministero della Salute RF-2019-12370896 to ALC and GDN.

For Peer Review Only

References

1. Go AS, Mozaffarian D, Roger VL*, et al*: Heart disease and stroke statistics--2014 update: a report from the American Heart Association. Circulation 2014, 129(3):e28-e292.
2. Expert Panel on Detection E, Treatment of High Blood Cholesterol in A: Executive Summary of The Third Report of The National Cholesterol Education Program (NCEP) Expert Panel on Detection, Evaluation, And Treatment of High Blood Cholesterol In Adults (Adult Treatment Panel III). Jama 2001, 285(19):2486-2497.
3. Conroy RM, Pyorala K, Fitzgerald AP*, et al*: Estimation of ten-year risk of fatal cardiovascular disease in Europe: the SCORE project. Eur Heart J 2003, 24(11):987-1003.
4. Wilson PW, Abbott RD, Castelli WP: High-density lipoprotein cholesterol and mortality. The Framingham Heart Study. Arteriosclerosis 1988, 8(6):737-741.
5. Emerging Risk Factors C, Di Angelantonio E, Sarwar N*, et al*: Major lipids, apolipoproteins, and risk of vascular disease. Jama 2009, 302(18):1993-2000.
6. Madsen CM, Varbo A, Nordestgaard BG: Extreme high high-density lipoprotein cholesterol is paradoxically associated with high mortality in men and women: two prospective cohort studies. Eur Heart J 2017, 38(32):2478-2486.
7. Zvintzou E, Karampela DS, Vakka A*, et al*: High density lipoprotein in atherosclerosis and coronary heart disease: Where do we stand today? Vascul Pharmacol 2021, 141:106928.
8. Voight BF, Peloso GM, Orho-Melander M*, et al*: Plasma HDL cholesterol and risk of myocardial infarction: a mendelian randomisation study. Lancet 2012, 380(9841):572-580.
9. Bonacina F, Pirillo A, Catapano AL, Norata GD: Cholesterol membrane content has a ubiquitous evolutionary function in immune cell activation: the role of HDL. Curr Opin Lipidol 2019, 30(6):462-469.
10. Norata GD, Catapano AL: Molecular mechanisms responsible for the antiinflammatory and protective effect of HDL on the endothelium. Vasc Health Risk Manag 2005, 1(2):119-129.
11. Zanotti I, Poti F, Cuchel M: HDL and reverse cholesterol transport in humans and animals: Lessons from pre-clinical models and clinical studies. Biochimica et biophysica acta Molecular and cell biology of lipids 2022, 1867(1):159065.
12. Kontush A, Lhomme M, Chapman MJ: Unraveling the complexities of the HDL lipidome. J Lipid Res 2013, 54(11):2950-2963.
13. Talbot CPJ, Plat J, Ritsch A, Mensink RP: Determinants of cholesterol efflux capacity in humans. Prog Lipid Res 2018, 69:21-32.
14. Rohatgi A, Khera A, Berry JD*, et al*: HDL cholesterol efflux capacity and incident cardiovascular events. N Engl J Med 2014, 371(25):2383-2393.
15. Saleheen D, Scott R, Javad S*, et al*: Association of HDL cholesterol efflux capacity with incident coronary heart disease events: a prospective case-control study. Lancet Diabetes Endocrinol 2015, 3(7):507-513.
16. Ebtehaj S, Gruppen EG, Bakker SJL, Dullaart RPF, Tietge UJF: HDL (High-Density Lipoprotein) Cholesterol Efflux Capacity Is Associated With Incident Cardiovascular Disease in the General Population. Arterioscler Thromb Vasc Biol 2019, 39(9):1874-1883.
17. Li XM, Tang WH, Mosior MK*, et al*: Paradoxical association of enhanced cholesterol efflux with increased incident cardiovascular risks. Arterioscler Thromb Vasc Biol 2013, 33(7):1696-1705.
18. Baragetti A, Norata GD, Sarcina C*, et al*: High density lipoprotein cholesterol levels are an independent predictor of the progression of chronic kidney disease. J Intern Med 2013, 274(3):252-262.
19. Baragetti A, Ossoli A, Strazzella A*, et al*: Low Plasma Lecithin: Cholesterol Acyltransferase (LCAT) Concentration Predicts Chronic Kidney Disease. J Clin Med 2020, 9(7).
20. Butler J, Shapiro M, Reiber J*, et al*: Extent and distribution of coronary artery disease: a comparative study of invasive versus noninvasive angiography with computed angiography. Am Heart J 2007, 153(3):378-384.

For Peer Review Only

21. Faletra FF, Klersy C, D'Angeli I, *et al*: Relation between coronary atherosclerotic plaques and traditional risk factors in people with no history of cardiovascular disease undergoing multi-detector computed coronary angiography. Heart 2009, 95(15):1265-1272.
22. Johnson KM, Dowe DA, Brink JA: Traditional clinical risk assessment tools do not accurately predict coronary atherosclerotic plaque burden: a CT angiography study. AJR Am J Roentgenol 2009, 192(1):235-243.
23. Silverman MG, Blaha MJ, Krumholz HM, *et al*: Impact of coronary artery calcium on coronary heart disease events in individuals at the extremes of traditional risk factor burden: the Multi-Ethnic Study of Atherosclerosis. Eur Heart J 2014, 35(33):2232-2241.
24. Magnoni M, Andreini D, Gorini M, *et al*: Coronary atherosclerosis in outlier subjects at the opposite extremes of traditional risk factors: Rationale and preliminary results of the Coronary Atherosclerosis in outlier subjects: Protective and novel Individual Risk factors Evaluation (CAPIRE) study. Am Heart J 2016, 173:18-26.
25. Austen WG, Edwards JE, Frye RL, *et al*: A reporting system on patients evaluated for coronary artery disease. Report of the Ad Hoc Committee for Grading of Coronary Artery Disease, Council on Cardiovascular Surgery, American Heart Association. Circulation 1975, 51(4 Suppl):5-40.
26. Magnoni M, Masson S, Andreini D, *et al*: Usefulness of High-Sensitivity Cardiac Troponin T for the Identification of Outlier Patients With Diffuse Coronary Atherosclerosis and Low-Risk Factors. The American journal of cardiology 2016, 117(9):1397-1404.
27. Andreini D, Conte E, Mushtaq S, *et al*: Plaque assessment by coronary CT angiography may predict cardiac events in high risk and very high risk diabetic patients: A long-term follow-up study. Nutrition, metabolism, and cardiovascular diseases : NMCD 2021, 10.1016/j.numecd.2021.11.013.
28. Morrow DA, Cannon CP, Jesse RL, *et al*: National Academy of Clinical Biochemistry Laboratory Medicine Practice Guidelines: Clinical characteristics and utilization of biochemical markers in acute coronary syndromes. Circulation 2007, 115(13):e356-375.
29. Cerda A, Hirata MH, Hirata RD: Molecular mechanisms underlying statin effects on genes involved in the reverse cholesterol transport. Drug Metabol Drug Interact 2012, 27(2):101-111.
30. Naresh S, Bitla AR, Rao P, Sachan A, Amancharla YL: Efficacy of oral rosuvastatin intervention on HDL and its associated proteins in men with type 2 diabetes mellitus. Endocrine 2021, 71(1):76-86.
31. Darabi M, Kontush A: High-density lipoproteins (HDL): Novel function and therapeutic applications. Biochimica et biophysica acta Molecular and cell biology of lipids 2022, 1867(1):159058.
32. Andreini D, Magnoni M, Conte E, *et al*: Coronary Plaque Features on CTA Can Identify Patients at Increased Risk of Cardiovascular Events. JACC Cardiovascular imaging 2020, 13(8):1704-1717.
33. Boden WE, Probstfield JL, Anderson T, *et al*: Niacin in patients with low HDL cholesterol levels receiving intensive statin therapy. N Engl J Med 2011, 365(24):2255-2267.
34. Barter PJ, Caulfield M, Eriksson M, *et al*: Effects of torcetrapib in patients at high risk for coronary events. N Engl J Med 2007, 357(21):2109-2122.
35. Schwartz GG, Olsson AG, Abt M, *et al*: Effects of dalcetrapib in patients with a recent acute coronary syndrome. N Engl J Med 2012, 367(22):2089-2099.
36. Lincoff AM, Nicholls SJ, Riesmeyer JS, *et al*: Evacetrapib and Cardiovascular Outcomes in High-Risk Vascular Disease. N Engl J Med 2017, 376(20):1933-1942.
37. Frikke-Schmidt R, Nordestgaard BG, Stene MC, *et al*: Association of loss-of-function mutations in the ABCA1 gene with high-density lipoprotein cholesterol levels and risk of ischemic heart disease. Jama 2008, 299(21):2524-2532.
38. Johannsen TH, Kamstrup PR, Andersen RV, *et al*: Hepatic lipase, genetically elevated high-density lipoprotein, and risk of ischemic cardiovascular disease. The Journal of clinical endocrinology and metabolism 2009, 94(4):1264-1273.
39. Haase CL, Tybjaerg-Hansen A, Grande P, Frikke-Schmidt R: Genetically elevated apolipoprotein A-I, high-density lipoprotein cholesterol levels, and risk of ischemic heart disease. The Journal of clinical endocrinology and metabolism 2010, 95(12):E500-510.
40. Holmes MV, Asselbergs FW, Palmer TM, *et al*: Mendelian randomization of blood lipids for coronary heart disease. Eur Heart J 2015, 36(9):539-550.

Email: IANN-peerreview@journals.tandf.co.uk URL: http://mc.manuscriptcentral.com/sann

For Peer Review Only

41. Lee JJ, Chi G, Fitzgerald C, *et al*: Cholesterol Efflux Capacity and Its Association With Adverse Cardiovascular Events: A Systematic Review and Meta-Analysis. Frontiers in cardiovascular medicine 2021, 10.3389/fcvm.2021.774418.
42. Catapano AL, Pirillo A, Bonacina F, Norata GD: HDL in innate and adaptive immunity. Cardiovasc Res 2014, 103(3):372-383.
43. Bonacina F, Coe D, Wang G, *et al*: Myeloid apolipoprotein E controls dendritic cell antigen presentation and T cell activation. Nat Commun 2018, 9(1):3083.
44. Gordon SM, Chung JH, Playford MP, *et al*: High density lipoprotein proteome is associated with cardiovascular risk factors and atherosclerosis burden as evaluated by coronary CT angiography. Atherosclerosis 2018, 278:278-285.
45. Salahuddin T, Natarajan B, Playford MP, *et al*: Cholesterol efflux capacity in humans with psoriasis is inversely related to non-calcified burden of coronary atherosclerosis. Eur Heart J 2015, 36(39):2662-2665.
46. Josefs T, Wouters K, Tietge UJF, *et al*: High-density lipoprotein cholesterol efflux capacity is not associated with atherosclerosis and prevalence of cardiovascular outcome: The CODAM study. J Clin Lipidol 2020, 14(1):122-132 e124.
47. Oshita T, Toh R, Nagano Y, *et al*: Association of cholesterol uptake capacity, a novel indicator for HDL functionality, and coronary plaque properties: An optical coherence tomography-based observational study. Clin Chim Acta 2020, 503:136-144.

For Peer Review Only

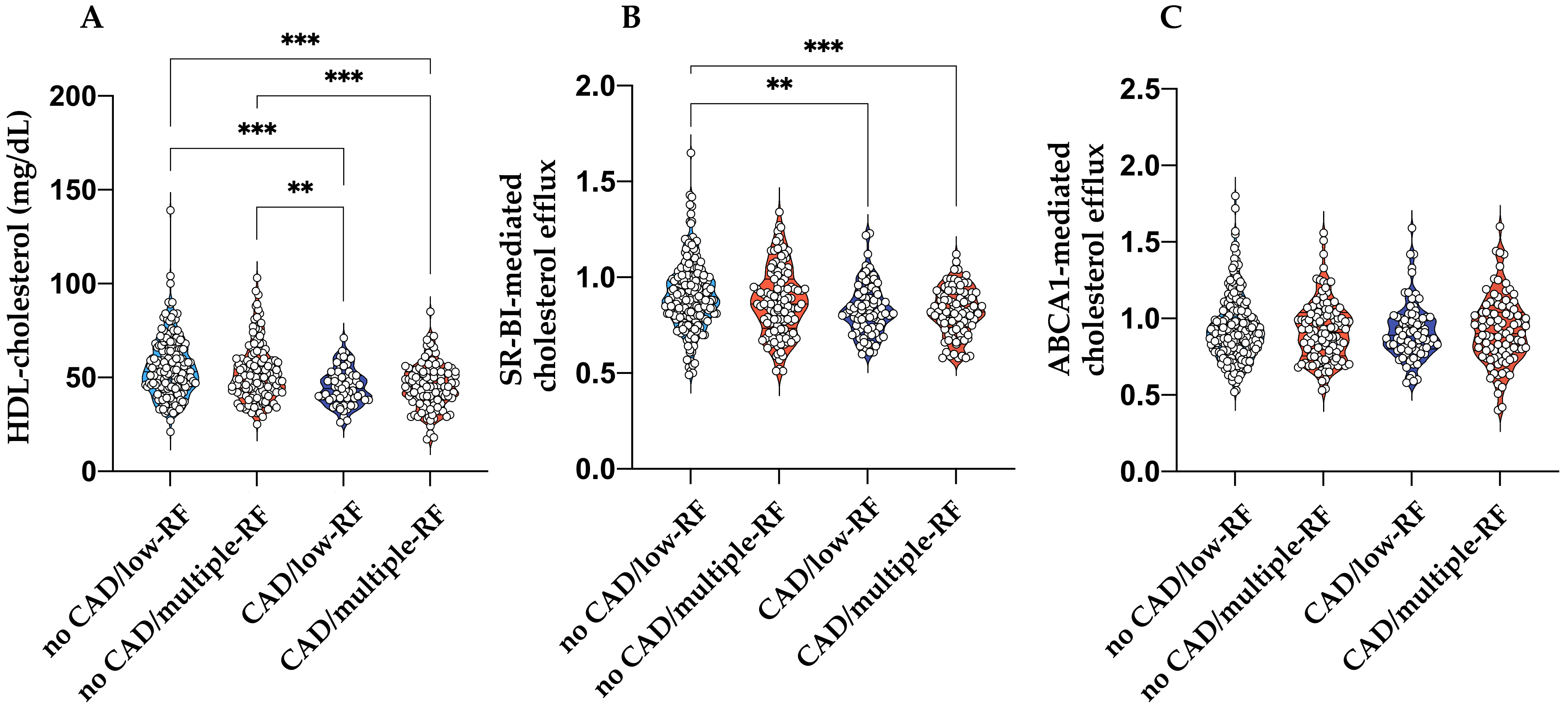
A
***
***
***
**
HDL-cholesterol (mg/dL)
200
150
100
50
0
no CAD/low-RF
no CAD/multiple-RF
CAD/low-RF
CAD/multiple-RF
B
***
**
SR-BI-mediated cholesterol efflux
2.0
1.5
1.0
0.5
0.0
no CAD/low-RF
no CAD/multiple-RF
CAD/low-RF
CAD/multiple-RF
C
ABCA1-mediated cholesterol efflux
2.5
2.0
1.5
1.0
0.5
0.0
no CAD/low-RF
no CAD/multiple-RF
CAD/low-RF
CAD/multiple-RF

Figure 1

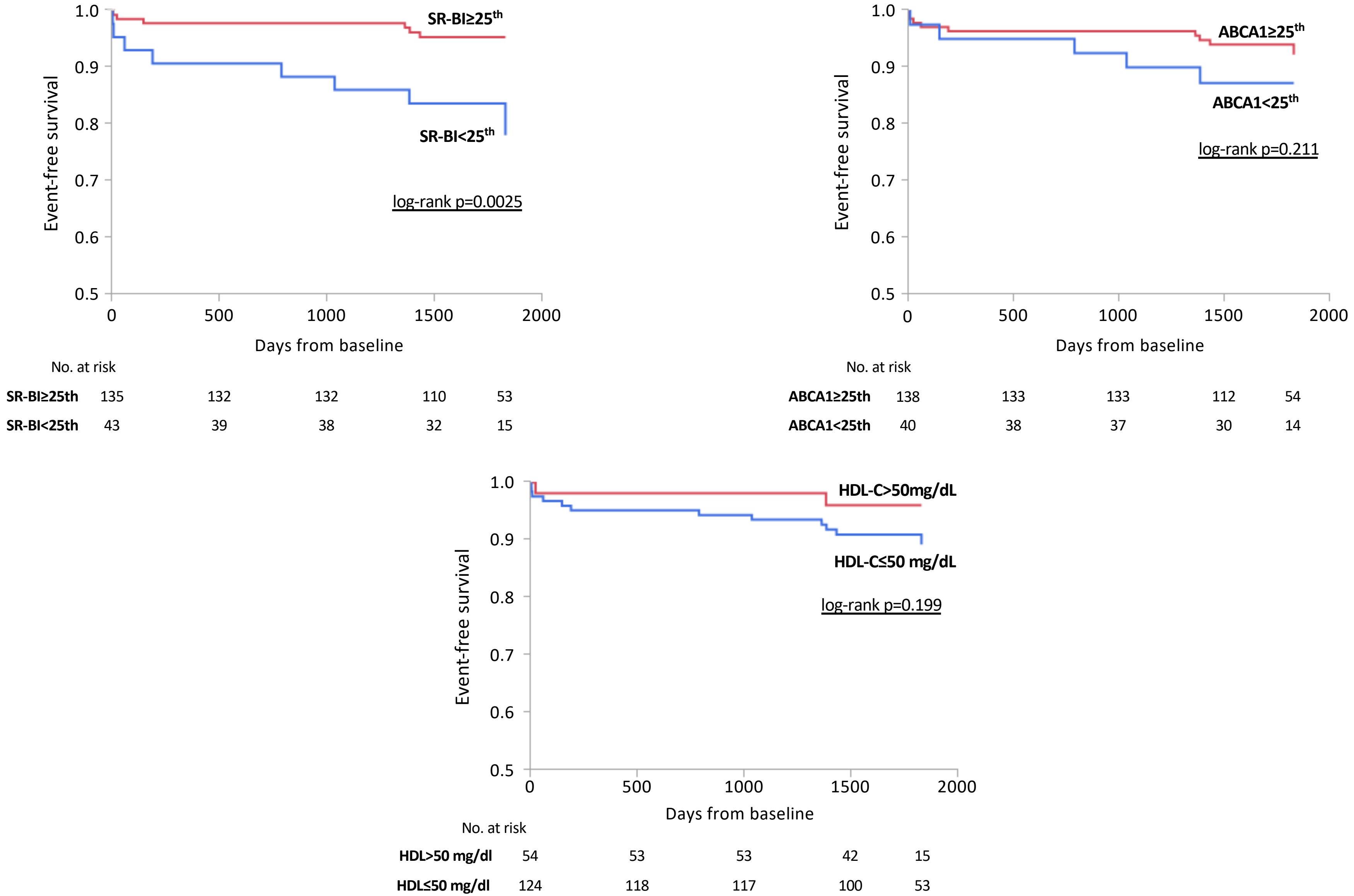
1.0
0.9
0.8
0.7
0.6
0.5
Event-free survival
SR-BI≥25th
SR-BI<25th
log-rank p=0.0025
0
500
1000
1500
2000
Days from baseline
No. at risk
SR-BI≥25th 135 132 132 110 53
SR-BI<25th 43 39 38 32 15
ABCA1≥25th
ABCA1<25th
log-rank p=0.211
No. at risk
ABCA1≥25th 138 133 133 112 54
ABCA1<25th 40 38 37 30 14
HDL-C>50mg/dL
HDL-C≤50 mg/dL
log-rank p=0.199
No. at risk
HDL>50 mg/dl 54 53 53 42 15
HDL≤50 mg/dl 124 118 117 100 53

Figure 2

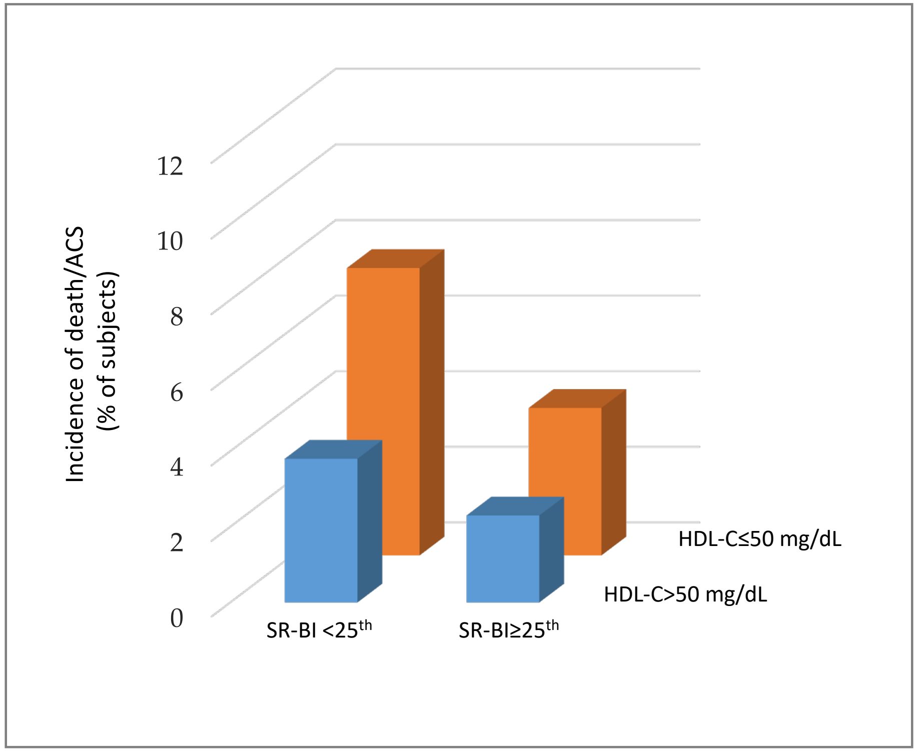
Incidence of death/ACS
(% of subjects)
12
10
8
6
4
2
0
SR-BI <25th
SR-BI≥25th
HDL-C≤50 mg/dL
HDL-C>50 mg/dL

Figure 3

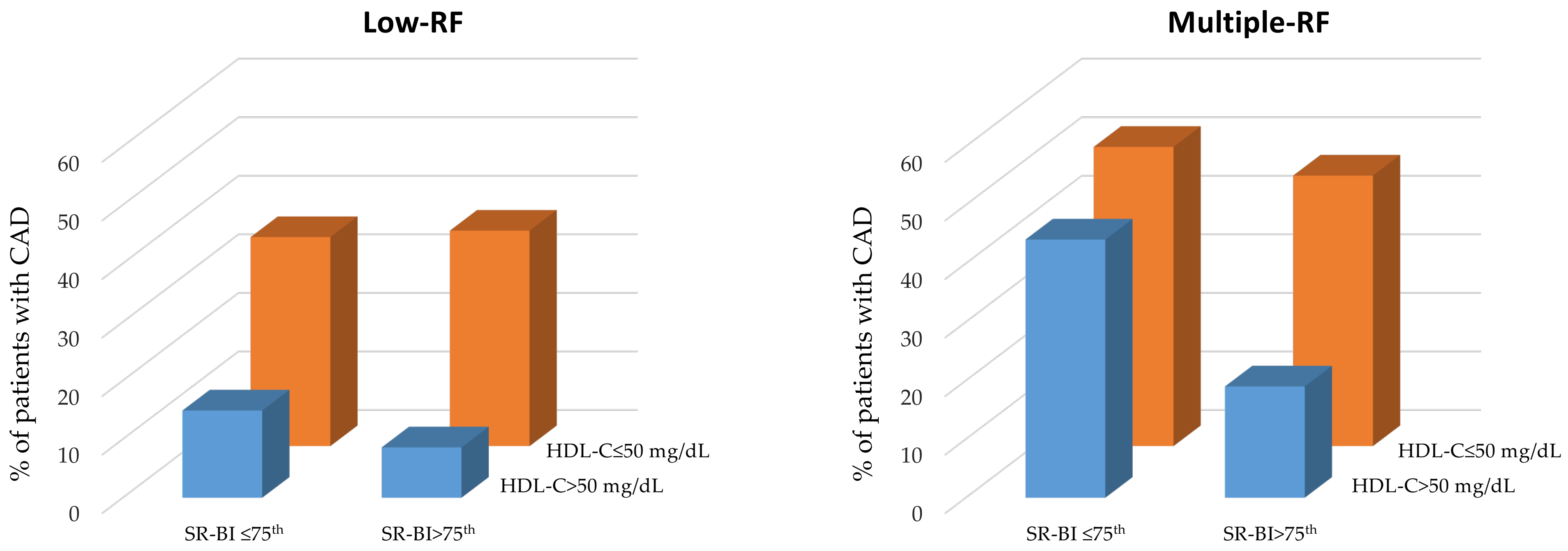

Supplementary Figure 1

Table 1. Baseline characteristics by risk factor (RF) number and coronary artery disease (CAD) categories

	**Low-RF (n=263)**			**Multiple-RF (n=262)**		
	**No-CAD (n=200)**	**CAD (SIS>5) (n=63)**	**p-value**	**No-CAD (n=147)**	**CAD (SIS>5) (n=115)**	**p-value**
Demographic						
Age, mean (SD), yrs	57.6 ± 8.6	63.6 ± 7.6	<0.0001	57.8 ± 8.1	62.9 ± 6.9	<0.0001
Male, %	49.5	93.7	<0.0001	42.2	73.9	<0.0001
BMI, mean (SD), Kg/m^2	25.1 ± 3.7	26.8 ± 4.1	0.0031	27.1 ± 4.0	28.3 ± 4.5	0.025
Medical history						
Family History of IHD, %	7.0	6.4	0.86	59.2	52.2	0.26
Arterial hypertension, %	21.0	31.8	0.09	77.6	86.9	0.06
Dyslipidemia, %	48	43	0.48	97.3	95.7	0.47
Current smoking, %	4.5	6.4	0.56	42.9	51.3	0.17
Diabetes, %	-	-	-	19.7	31.3	0.031
Systolic BP mean (SD), mmHg	125.1 ± 13.5	128.3 ± 13.4	0.13	128.1 ± 14.7	135.9 ± 17.3	0.004
Statin therapy, %	9	12.3	0.43	46.3	59.1	0.038
Laboratory data						
Total cholesterol, mean (SD), mg/dL	195.2 ± 39.3	188.0 ± 31.8	0.14	210.5 ± 44.5	188.7 ± 44.5	0.0001
LDL-cholesterol, mean (SD), mg/dL	119.3 ± 32.5	120.0 ± 28.9	0.86	128.7 ± 34.9	118.3 ± 37.4	0.0227
Triglycerides, median (IQR), mg/dL	73 (54-105)	104 (72-142)	<0.0001	111 (76-178)	115 (84-165)	0.62
HDL-cholesterol, mean (SD), mg/dL	55.2 ± 16.0	45.0 ± 9.7	<0.0001	52.5 ± 15.1	45.5 ± 12.1	<0.0001
SR-BI-mediated cholesterol efflux	0.90 ± 0.18	0.84 ± 0.14	0.008	0.88 ± 0.17	0.82 ± 0.12	0.002
ABCA1-mediated cholesterol efflux	0.93 ± 0.22	0.91 ± 0.19	0.48	0.92 ± 0.18	0.92 ± 0.21	0.97
C-reactive protein, median (IQR), mg/L	1.2 (0.5-2.7)	1.5 (0.5-4.6)	0.194	1.7 (0.8-4.4)	2.4 (0.9-5.8)	0.138
Serum creatinine, mean (SD), mg/dL	0.80 ± 0.16	0.91 ± 0.2	0.0003	0.80 ± 0.18	0.87 ± 0.18	0.004

RF: risk factor; CAD: coronary artery disease; SIS: segment involvement score; BMI: body mass index; IHD: ischemic heart disease; BP: blood pressure; LDL: low density lipoprotein; HDL: high density lipoprotein; SR-BI: scavenger receptor class B type I; ABCA1: ATP binding cassette transporter A1; IQR: interquartile range; SD: standard deviation.

For Peer Review Only

Table 2. Correlation between SR-BI (A) or ABCA-1 (B) cholesterol efflux capacity and the main continuous variables. Values (Rho) are Spearman's correlation coefficients.

**A**	**Overall**		**Low-RF**		**Multiple-RF**		**No-CAD**		**CAD**		**CAD/LRF**		**No CAD/MRF**	
	**Rho**	**p value**	**Rho**	**p value**	**Rho**	**p value**	**Rho**	**p value**	**Rho**	**p value**	**Rho**	**p value**	**Rho**	**p value**
Age, yrs	0.0042	0.9231	0.0644	0.2980	-0.0499	0.4214	0.1163	0.0303	-0.0414	0.5832	-0.0109	0.9326	0.0541	0.5149
BMI, kg/m^2	**-0.2376**	**<0.0001**	**-0.2691**	**<0.0001**	**-0.1509**	**0.0145**	**-0.2367**	**<0.0001**	-0.0958	0.2032	-0.2320	0.0673	**-0.1949**	**0.0180**
Systolic BP, mmHg	**-0.1530**	**0.0004**	-0.0986	0.1105	**-0.1836**	**0.0029**	-0.0886	0.0996	**-0.1531**	**0.0414**	-0.1650	0.1963	-0.1262	0.1277
Total cholesterol, mg/dL	**0.1228**	**0.0048**	0.1149	0.0629	**0.1472**	**0.0171**	0.0634	0.2391	**0.2005**	**0.0073**	0.1384	0.2795	-0.0092	0.9123
LDL-cholesterol, mg/dL	0.0445	0.3085	-0.0031	0.9606	0.1052	0.0893	-0.0348	0.5180	**0.1774**	**0.0178**	**0.4386**	**0.0003**	**0.5382**	**<.0001**
Triglycerides, mg/dL	**-0.2472**	**<0.0001**	**-0.2505**	**<0.0001**	**-0.1926**	**0.0017**	**-0.2443**	**<0.0001**	-0.1462	0.0515	0.0521	0.6851	-0.0504	0.5445
HDL-cholesterol, mg/dL	**0.4927**	**<0.0001**	**0.4603**	**<0.0001**	**0.5214**	**<0.0001**	**0.4872**	**<0.0001**	**0.4430**	**<0.0001**	-0.2364	0.0621	**-0.2687**	**0.0010**
C-reactive protein, mg/L	**-0.1129**	**0.0096**	**-0.1217**	**0.0486**	-0.0802	0.1957	-0.1032	0.0548	-0.0568	0.4513	-0.2308	0.0687	-0.1551	0.0607
IL-6, pg/ml	**-0.1169**	**0.0073**	**-0.1858**	**0.0025**	-0.0353	0.5696	-0.1013	0.0594	-0.1267	0.0919	**-0.2729**	**0.0305**	-0.0326	0.6946
Serum creatinine, mg/dL	**-0.2082**	**<0.0001**	**-0.1841**	**0.0027**	**-0.2399**	**<0.0001**	**-0.1699**	**0.0015**	**-0.1826**	**0.0147**	-0.1503	0.2395	**-0.1924**	**0.0195**

**B**	**Overall**		**Low RF**		**Multiple RF**		**No CAD**		**CAD**		**CAD/LRF**		**No CAD/MRF**	
	**Rho**	**p value**	**Rho**	**p value**	**Rho**	**p value**	**Rho**	**p value**	**Rho**	**p value**	**Rho**	**p value**	**Rho**	**p value**
Age. yrs	0.0310	0.4785	0.1345	0.0293	-0.0875	0.1577	0.0863	0.1085	-0.0423	0.5754	0.1322	0.3017	-0.0270	0.7453
BMI, kg/m^2	-0.0371	0.3960	-0.0268	0.6656	-0.0538	0.3854	-0.0519	0.3355	0.0014	0.9853	0.1578	0.2167	-0.0314	0.7060
Systolic BP, mmHg	0.0050	0.9088	0.0481	0.4370	-0.0307	0.6209	-0.0268	0.6182	0.0803	0.2869	0.1258	0.3260	-0.1081	0.1925
Total cholesterol, mg/dL	**0.3338**	**<0.0001**	**0.3109**	**<0.0001**	**0.3517**	**<0.0001**	**0.3695**	**<0.0001**	**0.2706**	**0.0003**	0.1787	0.1610	**0.4037**	**<0.0001**
LDL-cholesterol, mg/dL	**0.2470**	**<0.0001**	**0.2091**	**0.0006**	**0.2705**	**<0.0001**	**0.2621**	**<0.0001**	**0.2174**	**0.0036**	0.1224	0.3393	**0.2877**	**0.0004**
Triglycerides, mg/dL	0.0858	0.0495	**0.1482**	**0.0161**	0.0396	0.5228	0.0874	0.1041	0.0739	0.3268	0.1808	0.1561	0.0432	0.6032
HDL-cholesterol, mg/dL	**0.1963**	**<0.0001**	**0.2124**	**0.0005**	**0.1890**	**0.0021**	**0.2330**	**<0.0001**	0.1468	0.0505	0.1302	0.3092	**0.2173**	**0.0082**
C-reactive protein, mg/L	0.0039	0.9290	-0.0330	0.5947	0.0331	0.5942	-0.0222	0.6796	0.0611	0.4180	-0.0021	0.9869	-0.0126	0.8796
IL-6, pg/ml	**0.1300**	**0.0028**	0.1099	0.0752	**0.1400**	**0.0234**	**0.1550**	**0.0038**	0.0879	0.2431	0.1033	0.4204	**0.1967**	**0.0170**
Serum creatinine, mg/dL	**-0.1261**	**0.0038**	**-0.1296**	**0.0357**	-0.1201	0.0522	**-0.1379**	**0.0101**	-0.0997	0.1854	-0.1130	0.3780	-0.1394	0.0922

RF: risk factor; LRF: low risk factors; MRF: multiple risk factors; BMI: body mass index; BP: blood pressure; LDL: low density lipoprotein; HDL: high density lipoprotein; IL-6: interleukin-6

For Peer Review Only

Table 3. Baseline CCTA characteristics according to cholesterol efflux capacity distribution and HDL-C levels

	SR-BI Cholesterol Efflux Capacity				ABCA1 Cholesterol Efflux Capacity				HDL-cholesterol		
	<25° (n 43)	25-75° (n 90)	>75° (n 45)	P value	<25° (n 40)	25-75° (n 90)	>75° (n 48)	P value	≤ 50 mg/dl (n 124)	> 50 mg/dl (n 54)	P value
**CCTA characteristics**											
Lumen stenosis >70%, n (%)	17 (39.5)	38 (42.2)	25 (55.6)	0.243	18 (45)	46 (51.1)	16 (33.1)	0.1354	57 (46)	23 (42.6)	0.677
SSS, mean±SD	11.6 ±6.4	11.5 ± 6.0	11.9 ± 5.1	0.93	12.3 ±7.1	11.9 ± 5.8	10.5 ± 4.7	0.273	11.8 ±6.0	11.2 ± 5.4	0.504
SIS, mean±SD	7.6 ± 2.1	7.6 ± 2.0	7.6 ± 1.8	0.989	7.6 ± 2.0	7.7 ± 2.1	7.4 ± 1.6	0.627	7.6 ± 1.9	7.7 ± 2.0	0.826
Leaman CT-adapted score, mean±SD	10.3 ±4.0	10.8 ± 4.4	10.6 ±3.9	0.831	10.6 ±4.6	10.8 ± 4.0	10.2 ±4.2	0.700	10.9 ±4.1	10.0 ± 4.3	0.211
RI>1.4, n (%)	30 (69.8)	53 (58.9)	21 (46.7)	0.089	21 (52.5)	57 (63.3)	26 (54.2)	0.401	76 (61.3)	28 (51.9)	0.240
LAP, n (%)	29 (67.4)	56 (62.2)	32 (71.1)	0.569	25 (62.5)	62 (68.9)	30 (62.5)	0.668	86 (69.3)	31 (57.4)	0.123
NRS, n (%)	14 (32.6)	14 (23.5)	11 (24.4)	0.076	7 (17.5)	26 (28.9)	6 (12.5)	0.064	34 (27.4)	5 (9.3)	0.007
SC, n (%)	19 (44.2)	30 (33.3)	19 (42.2)	0.394	14 (35.0)	33 (36.7)	21 (43.7)	0.641	47 (37.9)	21 (38.9)	0.901
More than 2 HRF per type, n (%)	13 (30.2)	20 (22.2)	15 (33.3)	0.335	7 (17.5)	29 (32.2)	12 (25.0)	0.204	36 (29.0)	12 (22.2)	0.347
Pl Total Vol (mm^3), median (IQR)	204 (100-312)	171 (103-277)	170 (100-277)	0.823	174 (93-290)	180 (109-355)	162.5 (100-260)	0.510	180 (102-312)	163.5 (101.5-266)	0.328
Pl Vol HU<150 (mm^3), median (IQR)	45 (20-97)	31.5 (5-70)	29 (10.5-71.5)	0.261	27 (5-84)	40.5 (15-86)	29 (7.5-70)	0.346	40 (12.5-85.5)	25.5 (3.9-64.4)	0.066
Pl Vol HU<30 (mm^3), median (IQR)	3 (0-17)	2 (0-7.4)	2.6 (0-9.5)	0.481	2 (0-10)	3 (0-11)	2 (0-5)	0.470	3 (0-10)	1 (0-5)	0.086
Plaque length (mm), median (IQR)	46 (27-63)	40.5 (27.8-56)	46 (27.5-61)	0.500	48 (32-63)	45.5 (28.7-63)	38.7 (24-50)	0.041	43.5 (27-62)	39.5 (28-53)	0.306
Pl Vol HU<150/Plaque length	28.4 (12.8-39.8)	17.5 (6.6-30.2)	20.3 (9.0-31.9)	0.051	14.7 (4.8-35.1)	22.4 (12.3-30.3)	14.3 (7.2-37.9)	0.457	22.7 (11.1-37.2)	15 (6.6-27.4)	0.062
Pl Vol HU<30/Plaque length	0.10 (0-0.25)	0.05 (0-0.2)	0.04 (0-0.2)	0.51	0.03 (0-0.23)	0.06 (0-0.22)	0.05 (0-0.19)	0.638	0.07 (0-0.22)	0.03 (0-0.15)	0.130
Myocardial mass, mean±SD	115.7 ± 26.6	119.1 ± 23.8	121.1 ± 31.0	0.630	115.6 ± 23.6	117.8 ± 24.5	123.1 ± 31.6	0.383	118.7 ± 25.7	119.0 ± 28.1	0.938

For Peer Review Only

CCTA: coronary computed tomography angiography; SR-BI: scavenger receptor class B type I; ABCA1: ATP binding cassette transporter A1; HDL: high density lipoproteins; SSS: segment stenosis score; SIS: segment involvement score; RI: Remodeling index; LAP: low attenuation plaque; NRS: napkin ring sing; SC: spotty calcification; HRF: high risk features; Pl Vol: plaque volume; IQR: interquartile range; SD: standard deviation

For Peer Review Only

Manuscript **ID SANN-2022-OR-0116**
"Predictive value of HDL function in patients with coronary artery disease: relationship with coronary plaque characteristics and clinical events"

Reviewer: 1

Comments to the Author

Level of HDL-C is not a strong predictor of ATCVD based on several failed drug trials aiming to elevate HDL-C and reduce risk as well as based on Mendelian randomization studies regarding gene variants associated with increased HDL levels. Therefore a shift from HDL-C hypothesis to HDL functionality is getting strong support. This is also the aim in the present study where the authors have using CAPIRE study subjects investigated how HDL function in SR-BI/ABCA1 cholesterol efflux process is associated with CAD and coronary artery characteristics (evaluated via CCTA analysis) and the incidence of MI. Two study groups were established based on low/high risk factor numbers with no CAD and CAD. CCTA data evaluated using segment involvement screre, SIS as S=0 and SIS>5. Major outcomes indicated that reduction of SR-BI efflux was significantly associated with increase in clinical events and deaths+ACS were elevated in those patients who had SR-BI efflux <25th percentile+HDL-C <50 mg/dL. In addition, SR-BI efflux has no association with plaque characteristics or elevated total, non-calcified and low-alternation plaque volume. Importantly, subjects with NCP>80mm3 + SR-BI efflux <25th percentile were significantly associated with deaths +ACS. The paper is clearly written and the methods used are generally relevant to test the aims of the study. The follow-up time used was 5 yrs among the subjects while CAPIRE project has 10 yr follow-up period. There are several issues, however, remaining that need further clarifications.

Dear Reviewer thank you for the positive evaluation of our work. In this revised version of the manuscript, we have addressed all aspects raised by you and by reviewer 2.

Please find below the replies to your comments.

MAJOR COMMENTS

1. The authors were using Fu5AH cell - model that is derived from rat hepatoma. Since the cell model is originating from liver cells rat hepatoma cell line Fu5AH has the unusual property of accumulating massive amounts of cholesteryl ester upon incubation with serum since SR-BI has dual function, i.e. participation in selective uptake of cholesterol esters from HDL as well as in cholesterol efflux. This could be one bias-causing effect. The authors probably did not measure cholesterol net entry to these cells? In addition, previous studies have shown that HDL associated phospholipid content

(Fournier et al. ATVB 1997) is a major determinant in cholesterol efflux potential from these cells. These should be discussed by the authors.

Your point is well taken. We must acknowledge that SR-BI has a dual function, as it mediates both free cholesterol efflux to HDL and the selective uptake of cholesteryl esters from HDL (but also other lipoproteins). Cholesterol-loaded Fu5AH hepatoma cell line represents an established method for the evaluation of SR-BI-mediated cholesterol efflux and we have not evaluated cholesteryl ester uptake in these cells.

Of note, we have performed the experiments in the presence of an ACAT inhibitor to prevent the conversion of free cholesterol into cholesteryl esters, thus maximizing the chances for promoting its efflux. Indeed when we measured ^{3}H-cholesterol liquid scintillation counting in cholesterol loaded cells (24h treatment with DMEM containing 5% FCS, ^{3}H-cholesterol (1 μCi/ml), and 2 μg/ml ACAT inhibitor Sandoz 58-035, followed by for 4 hours of incubation in serum-free medium), and compared this condition with that of cells treated with serum, a clear reduction in intracellular cholesterol levels and an increase in extracellular cholesterol was observed, pointing toward a contribution of SR-BI mainly oriented toward cholesterol efflux under these experimental conditions (see below).

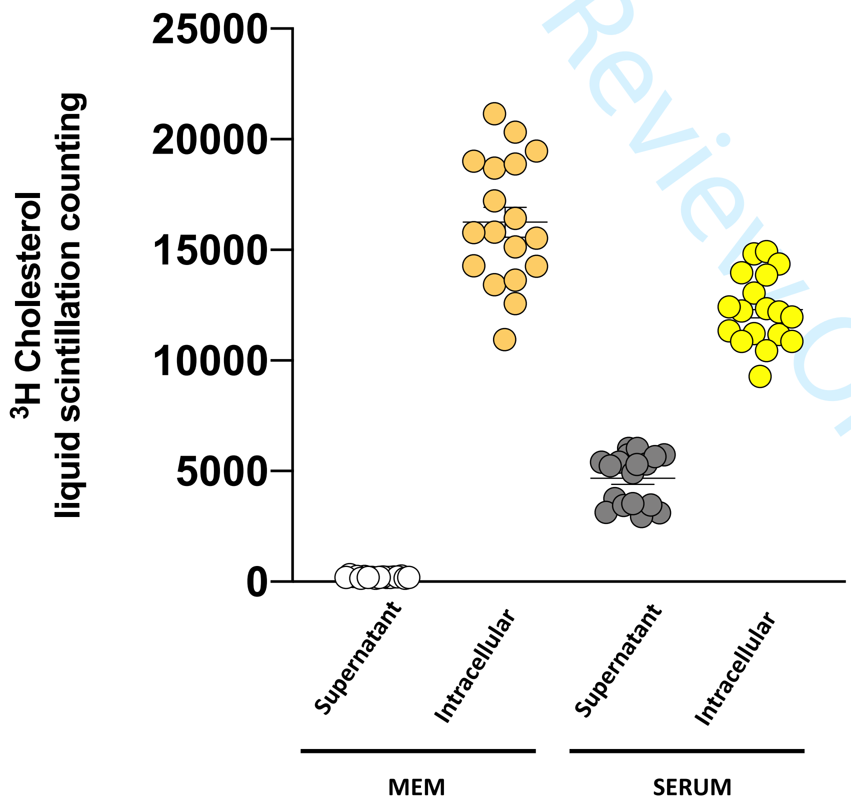

Although we acknowledge that HDL composition, including its phospholipid content, may influence significantly HDL ability to promote cholesterol efflux, the investigation of the characteristics of HDL (changes in size and composition) was beyond the scope of this

For Peer Review Only

study. We agree that a more in-depth investigation of the distribution of HDL subspecies and their lipid/protein composition in patients from this study is warranted.
According to your comment, in the revised manuscript we have mentioned the dual role of SR-BI and the relevance of HDL phospholipid content/composition in HDL-mediated cholesterol efflux. (see Introduction, page 4).

2. The authors do not show data on Lp(a) in these patients? Why? Earlier total serum CEC was shown to be reduced in patients with high Lp(a) levels due to inhibition of plasminogen-mediated ABCA1 cholesterol efflux by Lp(a) (Tavori et al. JCEM 2019; Pamir et al. JCI Insight 2017).

Your point is well taken. Lp(a) has been measured in a subset of CAPIRE patients (see the table below for the results):

	Low RF (n=31)			Multiple RF (n=46)		
	No CAD (n=18)	CAD (SIS>5) (n=13)	p-value	No CAD (n=22)	CAD (SIS>5) (n=24)	p-value
Lp (a), median (IQR)	12.1 (6.5-22.9)	10.5 (3.5-28.7)	0.88	11.1 (3.4-25.1)	16.1 (4.2-59.7)	0.33

We have also investigated the correlation between Lp(a) levels and SR-BI- or ABCA-1-mediated CEC; no significant correlations were found (Lp (a) vs ABCA1, Rho 0.052 p=0.65; Lp (a) vs SR-BI, Rho -0.0013 p=0.99) (see below).

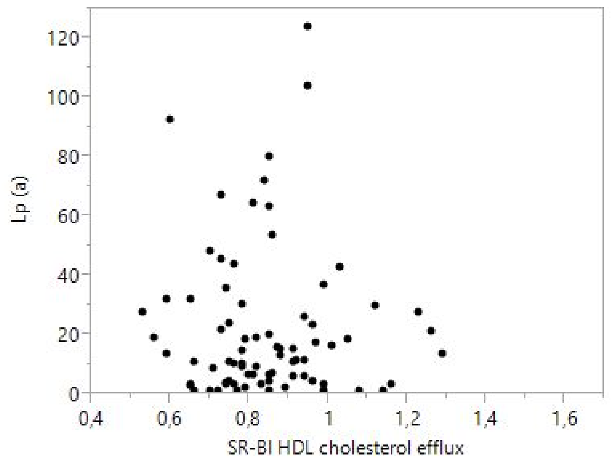

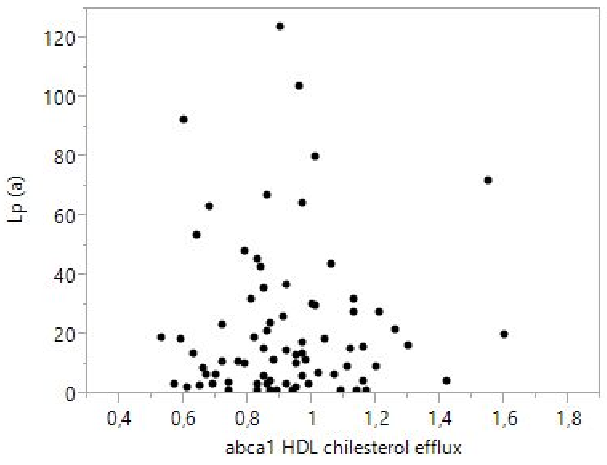

3. The authors measured ABCA1 cholesterol efflux using macrophage J774 model. Since macrophage expressed SR-BI is also participating in cholesterol efflux and macrophage-foam cells are the relevant ones compared to liver derived cells, the reviewer wonders why this SR-BI efflux was not measured in more relevant macrophage model? Of course have to make a word of caution that the role of SR-BI is more ambivalent and probably relates to metabolic/inflammatory status.

For Peer Review

Thank you for raising this point. We fully agree that SR-BI is expressed also in macrophages and could play a relevant role in cholesterol efflux during atherogenesis. However, for this study, we used a well-established model of ABCA1-mediated cholesterol efflux, which involves the use of cholesterol-loaded J774 macrophages expressing a high amount of ABCA1 following stimulation with 8-Br-cAMP. Under these experimental conditions, the expression of SR-BI in J774 cells loaded with AcLDL (see methods) is highly reduced, suggesting a minimal involvement of this receptor in cholesterol efflux (https://doi.org/10.1194/jlr.M300461-JLR200). To evaluate the SR-BI-mediated CEC, on the other hand, we used Fu5AH hepatoma cell line, which basally expresses a high amount of SR-BI.

4. The authors measured cholesterol efflux potential using whole serum as cholesterol acceptor from the radioactive-cholesterol labelled cells. Why they did not utilize apoB-depleted serum that has been broadly used in those studies demonstrating the association of elevated cholesterol efflux with reduced risk of CVD?

Thank you for raising this point. There are two main reasons why the experiments were performed using the whole serum. The first is that different apoB precipitation methods may have an impact on HDL, including changes in HDL size profile, composition, or both, and may thus alter HDL function, including its cholesterol efflux capacity (for a reference, see https://doi.org/10.1194/jlr.M066613). The second reason is that serum represents a more "physiological condition" since, in vivo, cells are exposed to a mixture of lipoproteins that participate in the net movement of cholesterol, thus allowing to evaluate the cholesterol efflux capacity as a whole. Several studies have been published in which cholesterol efflux capacity has been evaluated in the whole serum, which thus represents a valuable method for assessing changes in this critical parameter. We must also highlight that, while ABCA1-mediated cholesterol efflux can be attributed almost exclusively to lipid-poor preß-HDL particles, SR-BI-mediated cholesterol is a more complex process, as this pathway may involve different subpopulations of HDL (and also other lipoproteins).

5. What was the intra- and inter-assay CV for SR-BI efflux assays?

Your point is well taken, intra- and inter-assay coefficients of variation for SR-BI-mediated cholesterol efflux were 4.7% and 14.3%, respectively. This is now stated in the materials and methods section.

6. Some of the statins used show higher anti-inflammatory effect. Was this issue considered in the study and possibly affecting the data outcome?

For Peer Review Only

Thank you for raising this aspect; we investigated the impact of statin treatment on inflammatory markers (CRP and IL-6) in the CAPIRE study. Overall, statin treatment did not affect either CRP and IL-6 plasma levels

	Statin no	Statin yes	
CRP, median (IQR), mg/L	1.4 (0.7-3.9)	1.7 (0.8-4.2)	0.164
IL-6, median (IQR),	7.6 (6.5-9.7)	7.7 (6.2-9.7)	0.99

In addition, we investigated whether specific differences could be appreciated in subgroups of patients; no major differences were observed apart from lower CRP plasma levels in the noCAD/LRF in the statin group (please note that only 9% of patients were on statins in this group, see table 1). These data are presented below for your evaluation.

	noCAD/LRF	CAD/LRF	noCAD/MRF	CAD/MRF
**CRP**				
Statin no	1.3 (0.6-2.9)	1.4 (0.5-5.1)	1.5 (0.7-4.5)	1.5 (0.8-6.0)
Statin yes	0.8 (0.3-1.5)	1.8 (0.5-3.4)	1.7 (1.0-4.0)	2.6 (1.0-5.7)
p value	0.02	0.63	0.76	0.49
**IL-6**				
Statin no	7.5 (6.3-9.7)	7.9 (6.2-10.1)	7.6 (6.7-9.5)	7.6 (6.7-9.1)
Statin yes	6.6 (5.8-7.6)	7.2 (6.4-9.3)	7.7 (5.8-9.6)	8.4 (6.4-9.3)
p value	0.058	0.8	0.69	0.39

7. Statin use was reported in the four study groups. Reportedly statins can affect cholesterol efflux process as such but also affecting HDL particles and improving both quantitative and qualitative properties of HDL particles; see for instance Naresh et al. Endocrine 2021. The authors should further discuss this point.

Your suggestion is well taken. We have analyzed the impact of statins on either SR-BI- and ABCA1-mediated cholesterol efflux; we did not observe changes in cholesterol efflux capacity in statin-treated patients compared to their counterparts We now discussed how statins can impact HDL particles and properties.

8. What might be the explanation to the observation here that ABCA1 facilitated efflux did not differ between no-CAD/low RF and CAD/multiple RF groups?

This finding was not surprising. In fact, it has been reported that patients with CAD have a HDL subfraction profile characterized by lower levels of larger particles (mainly involved in SR-BI-mediated cholesterol efflux) but unchanged or even higher levels of pre-ß-particles (Am J Cardiol. 2003 Apr 3;91(7A):12E-17E. doi: 10.1016/s0002-9149(02)03383-0. High-density

For Peer Review Only

lipoprotein subpopulations in pathologic conditions. (Bela F Asztalos 1, Ernst J Schaefer), which remove cholesterol upon interaction with ABCA1. This observation can explain the lack of differences in ABCA1-mediated CEC in the different subgroups of patients enrolled in this study.

9. Limitations addressed in the Discussion are relevantly targeted in the context of this study.

We appreciate your comment and incorporated the comments above within the text.

For Peer Review Only

Reviewer: 2

Comments to the Author

The manuscript by Norata and colleagues is an interim analysis of the clinical trial CAPIRE aimed at investigating association and prognostic value of cholesterol efflux capacity in patients with few (0-1) or multiple (≥2) cardiovascular risk factors (CVRF) with or without coronary artery disease (CAD, assessed by coronary CT scan). After a median follow-up of 5 years, the authors have found that low SRB1-mediated cholesterol efflux (i.e., lower quartile) was associated with a higher rate of cardiovascular events. Moreover, lower SRB1-mediated was associated with the prevalence of CAD in cross-sectional analyses but not with changes in radiological features of high-risk atheromas. Conversely, no significant association was found with ABCA1-mediated cholesterol efflux. The authors conclude that SRB1-mediated cholesterol efflux may be associated with worse clinical outcomes in patients with CAD, independently of plaque phenotype.

The study is interesting as it addresses the intriguing clinical question of the potential relevance of cholesterol efflux in the development of CAD also in patients with no established risk factors. The manuscript is well written, the clinical trial is properly conducted, and conclusions are substantially supported by the results.

Dear Reviewer thank you for the positive evaluation of our work. In this revised version of the manuscript, we have addressed all aspects raised by you and by reviewer 2.

Please find below the replies to your comments.

1) Page 7: the authors enlist the risk factors considered in the study. The criteria for the definition of these risk factors should be included (e.g., was hypertension defined as BP>140/90 or also as concomitant anti-hypertensive treatment? How was hypercholesterolemia defined?).

Your point is well taken. Following is a detailed description of the risk factors taken into account: family history of CAD (history of early manifestations of CAD in first-degree relatives, <55 years old for men and <65 years old for women), systemic hypertension (history of arterial hypertension, ongoing antihypertensive treatment, or recent observation of blood pressure values >140/90 mmHg), hypercholesterolemia (total cholesterol >200 mg/dl or <200 mg/dl if under lipid-lowering therapy), diabetes mellitus (fasting plasma blood glucose levels >126 mg/dL, or 2-hour values in the oral glucose tolerance test ≥200 mg/dL, or isolated elevation of glycated hemoglobin ≥6.5%, or current use of insulin or oral hypoglycemic agents) [24]. These data were originally reported in the submission, but the Editorial Office asked us to include a reference to the inclusion criteria without mentioning them again in the material and methods section. We have now included again this part in the manuscript.

For Peer Review Only

2) This reviewer finds interesting the lack of association between ABCA1-dependent cholesterol efflux and clinical outcomes. It has been reported that statins (e.g., pmid: 31409515, 27989886, and others) or anti-diabetic drugs (e.g., pmid: 28526884, 32667970, and others) may affect ABCA1 expression (and function) and table 1 shows as diabetes and statin may be more common in patients with CAD. Did the authors find any difference in the prognostic power of ABCA1-dependent cholesterol efflux in patients without diabetes or statin treatment?

Your suggestion is well taken; we investigated the prognostic power of differences in ABCA-1 dependent cholesterol efflux on primary outcome in patients without statins as well as in patients without diabetes but no significant differences in the primary outcome were observed.

	Overall			no diabetes			no statin		
Variable	HR	95%CI	p value	HR	95%CI	p value	HR	95%CI	p value
ABCA1-mediated CEC									
> 25th percentile	Ref.			Ref.			Ref		
<25th	2.05	0.63-5.96	0.22	2.0	0.42-7.6	0.35	2.34	0.49-9.09	0.26

3) Page 11: the authors may consider reporting the number of MACEs observed during the follow-up in the whole cohort and in the different quartiles. Moreover, the "population at risk" may be reported in the Kaplan-Meier curves in Figure 2

Your point is well taken, the number of MACEs observed during the follow-up in the whole cohort and in the different quartiles was the following: the number of MACEs in the entire cohort was 21 distributed among the group LR-No CAD 2; LR-CAD 2; HR-NO CAD 5; HR-CAD 12. These data are now in the result section. Figure 2 has been revised and the population at risk in each panel has been introduced.

4) Figure 1: the data are apparently analyzed by one-way ANOVA. If the statistical interaction among the two factors (i.e., number of CVRF and prevalence of CAD) is of interest, the authors may consider analyzing these data by factorial ANOVA and report also a P-value for the interaction (e.g., in the figure legend). Moreover, the post hoc test for pairwise comparison following ANOVA should be reported in the "statistical analysis" paragraph (page 9).

Your point is well taken. Tukey post-hoc analysis for pairwise comparison following two ways ANOVA test was performed. Also, factorial analysis has been performed and P-values for interaction are reported below:

For Peer Review Only

2way ANOVA<br>ANOVA results				
Table Analyzed	Per interaction SRBI			
**Two-way ANOVA**	Ordinary			
Alpha	0.05			
**Source of Variation**	**% of total variation**	**P value**	**P value summary**	**Significant?**
Interaction	0.1118	0.4358	ns	No
RF	0.3245	0.1845	ns	No
Presence of CAD	3.008	<0.0001	****	Yes

For Peer Review Only
